# DNA Methylation Analysis of Imprinted Genes in the Cortex and Hippocampus of Cross-Fostered Mice Selectively Bred for Increased Voluntary Wheel-Running

**DOI:** 10.1007/s10519-022-10112-z

**Published:** 2022-08-21

**Authors:** Sarah E. Latchney, Marcell D. Cadney, Austin Hopkins, Theodore Garland

**Affiliations:** 1 Department of Biology, St. Mary’s College of Maryland, 18952 E. Fisher Rd, Saint Mary’s City, MD 20686 USA; 2 Department of Evolution, Ecology, and Organismal Biology, University of California, Riverside, CA 92521 USA; 3 EpigenDx, Inc, Hopkinton, MA 01748 USA

**Keywords:** Bisulfite sequencing, Exercise, Cross-fostering, Maternal effects, Parent-of-origin genes, Brain

## Abstract

We have previously shown that high runner (HR) mice (from a line genetically selected for increased wheel-running behavior) have distinct, genetically based, neurobiological phenotypes as compared with non-selected control (C) mice. However, developmental programming effects during early life, including maternal care and parent-of-origin-dependent expression of imprinted genes, can also contribute to variation in physical activity. Here, we used cross-fostering to address two questions. First, do HR mice have altered DNA methylation profiles of imprinted genes in the brain compared to C mice? Second, does maternal upbringing further modify the DNA methylation status of these imprinted genes? To address these questions, we cross-fostered all offspring at birth to create four experimental groups: C pups to other C dams, HR pups to other HR dams, C pups to HR dams, and HR pups to C dams. Bisulfite sequencing of 16 imprinted genes in the cortex and hippocampus revealed that the HR line had altered DNA methylation patterns of the paternally imprinted genes, *Rasgrf1* and *Zdbf2*, as compared with the C line. Both fostering between the HR and C lines and sex modified the DNA methylation profiles for the paternally expressed genes *Mest*, *Peg3, Igf2, Snrpn,* and *Impact*. *Ig-DMR*, a gene with multiple paternal and maternal imprinted clusters, was also affected by maternal upbringing and sex*.* Our results suggest that differential methylation patterns of imprinted genes in the brain could contribute to evolutionary increases in wheel-running behavior and are also dependent on maternal upbringing and sex.

**Supplementary information:**

The online version contains supplementary material available at 10.1007/s10519-022-10112-z.

## Introduction

Exercise has powerful effects on neurobiological processes such as neurotransmission, cognition, and reward-dependent behaviors (Rhodes et al. 2005; Caetano-Anolles et al. 2016; Saul et al. 2017). While motivation and physiological ability are critical determinants of an individual’s physical activity levels (Garland et al. 2017), long-term genetic selection experiments in laboratory mice have also illuminated genes associated with elevated levels of voluntary exercise (Kelly et al. 2012; Kostrzewa and Kas 2014; Vellers et al. 2018; Aasdahl et al. 2021). RNA (Zhang et al. 2018) and whole genome (Xu and Garland 2017; Hillis et al. 2020) sequencing from mice selected for elevated physical activity levels have revealed differential expression of genes associated with behavior, motivation, and athletic ability, supporting a genetic basis for elevated physical activity. In addition to genetic underpinnings, non-genomic, developmental programming effects during early life can also substantially impact exercise behavior and physical activity (Li et al. 2013; Baker et al. 2015; Eclarinal et al. 2016; Garland et al. 2017).

One such developmental programming mechanism involves genomic imprinting. Unlike standard Mendelian inheritance patterns where genes are biallelically expressed, genomically imprinted genes display parent-of-origin and monoallelic expression (Bartolomei and Ferguson-Smith 2011; Barlow and Bartolomei 2014). Approximately 1% of all mammalian genes are thought to be imprinted, with about 150 imprinted genes identified to date (Barlow and Bartolomei 2014; Kalish et al. 2014). Imprinted genes are critical for growth and development (Bouschet et al. 2017; Monk et al. 2019), cognition (Isles and Wilkinson 2000; Franklin and Mansuy 2010; Zamarbide et al. 2018), and exercise (Kelly et al. 2010). Parent-of-origin effects occur through several molecular mechanisms. The most well studied mechanism is differential DNA methylation (Bartolomei and Ferguson-Smith 2011; Barlow and Bartolomei 2014). The parental origin frequently determines the methylation status, with the paternal or maternal genomes typically exerting counteracting influences on gene expression (Azzi et al. 2014; Barlow and Bartolomei 2014; Kalish et al. 2014). For example, paternally expressed genes (PEG) such as *Igf2* and *Mest* generally enhance growth of the fetus, while maternally expressed genes (MEG) such as *H19* and *Igf2r* inhibit fetal growth (DeChiara et al. 1990; Leighton et al. 1995; Thorvaldsen et al. 1998). Moreover, differentially methylated regions exhibit variable methylation patterns following exposure to various early-life conditions such as diet (Waterland et al. 2006; Kovacheva et al. 2007; Heijmans et al. 2008; Tobi et al. 2009; Hoyo et al. 2011) and chemical exposure (Robles-Matos et al. 2021). Therefore, the methylation profile of imprinted genes is an appealing target to investigate in the context of exercise behavior because the expression or silencing of these monoallelic genes is critically involved in growth regulation and energy balance during development (Charalambous et al. 2007; Tunster et al. 2013; Waterland 2014; Millership et al. 2019) which can influence brain function (Perez et al. 2016; Kravitz and Gregg 2019).

The developing brain is a known hotspot for allele-specific gene expression (Perez et al. 2016; Huang et al. 2017, 2018; Kravitz and Gregg 2019). Imprinted genes in the brain encode various proteins and non-coding RNAs, from ubiquitination-related proteins to neurotransmitter receptor subunits. Similar to their counteracting effects on body size, imprinted genes have reciprocal effects on brain size, with MEGs enhancing and PEGs reducing brain size, supporting a role for imprinted genes in neurodevelopment (Keverne et al*.* 1996, Bouschet et al. 2017). The most substantial evidence for a neurodevelopmental role in humans emerged when Angelman syndrome (AS) and Prader-Willi syndrome (PWS) were linked to maternally and paternally transmitted mutations, respectively, on human 15q11-13. In the case of AS, *UBE3A* is a MEG located among a cluster of imprinted genes on chromosome 15 (Albrecht et al. 1997; LaSalle et al. 2015), and maternally inherited mutations or deletions in *UBE3A* lead to AS (Cassidy et al. 2000). *UBE3A* imprinting is regulated by the expression of an antisense transcript, UBE3A-ATS, which arises from a nearby imprinting control region that is methylated only on the maternal allele. The UBE3A-ATS transcript partially overlaps the UBE3A gene, blocking UBE3A expression from the paternal allele (Albrecht et al. 1997; Yamasaki et al. 2003; Chamberlain and Lalande 2010). Reciprocal mutations similar to those of AS cause PWS due to the lack of PEG products in the 15q11-13 chromosomal region (Cassidy et al. 2000). A neurodevelopmental role for genomic imprinting is supported further by the widespread expression of imprinted genes in the mouse brain and by the extensive neural and behavioral phenotypes of mutants of these imprinted genes (Babak et al. 2008; Wang et al. 2008; Gregg et al. 2010; DeVeale et al. 2012; Bouschet et al. 2017) including *Cdkn1c* (Imaizumi et al. 2020; Laukoter et al. 2020), *Gnas* (Mouallem et al. 2008; Turan and Bastepe 2013), *Sgce* (Zimprich et al. 2001; Asmus et al. 2002; Peall et al. 2013), and *Trappc9* (Mochida et al. 2009; Babak et al. 2015).

Given the prominent roles of monoallelically expressed genes in growth, development, and energy metabolism in the brain, this study aimed to distinguish DNA methylation from genetic contributions for elevated physical activity in brains of mice that have been genetically selected for increased wheel-running behavior. In a long-term genetic selection experiment using outbred Hsd:ICR mice, four replicate high runner (HR) lines were bred for high voluntary wheel-running behavior and four control (C) lines were bred without regard to their wheel-running (C mice; Swallow et al. 1998, 1999). Compared with C lines, HR lines run ~ threefold more daily revolutions on running wheels. In addition, HR mice also demonstrate physiological and biochemical adaptations that are consistent with increased physical activity, including smaller body size, decreased fat composition, altered lipid profiles, and increased aerobic capacity (Swallow et al. 1999; Malisch et al. 2007; Acosta et al. 2015). More recently, early-life studies on HR mice demonstrated differential responses to early-life factors of altered juvenile diet and exercise opportunity (Acosta et al. 2015; Hiramatsu et al. 2017; Cadney et al. 2021a; McNamara et al. 2021).

In the current experiment, four groups were created wherein the offspring of non-selected C mice were fostered at birth and were reared by either a C or HR dam. Similarly, the offspring of selected HR mice were fostered at birth such that they were raised either by a C or HR dam (Cadney et al. 2021b). Using this cross-fostering approach, the current study sought to address two questions. First, do mice from a line genetically selected for elevated voluntary physical activity have altered DNA methylation profiles of imprinted genes compared to non-selected C mice? Second, does maternal upbringing further modify the DNA methylation status of these imprinted genes? Cross-fostering experiments are a powerful method to determine maternal effects and possible gene × environment interactions (Fish et al. 2004; Weaver et al. 2004; Kessler et al. 2011; Cohen et al. 2015; McCarty 2017). Therefore, to address these questions, we used cross-fostered HR and C male and female offspring from generation 90 of the HR selection experiment (see Cadney et al. 2021b for further methodological details concerning focal mice and their birth and foster parents). Using bisulfite sequencing of 16 known imprinted genes, we demonstrate altered DNA methylation patterns in the cortex and hippocampus for imprinted genes with known roles in fetal growth and energy metabolism. Our results show for the first time that, in addition to genetic underpinnings, differential methylation patterns of imprinted genes may also contribute to increased wheel-running behavior.

## Methods

### Experimental mice

The artificial selection experiment began in 1993 with a population of 224 mice from the outbred Hsd:ICR strain, which was randomly mated for two generations before being randomly partitioned into eight lines. Four replicate high runner (HR) lines of house mice were bred in the ongoing selection experiment for high voluntary wheel-running, based on average wheel revolutions per day on days five and six of a six-day running period as young adults. Four additional lines were bred randomly as Control (C) lines to the four HR lines (Swallow et al. 1998; Cadney et al. 2021b). The previous experiment used a subset of virgin male and female mice from generation 89 to produce experimental mice of generation 90 in the prior study. All mice were fed standard mouse chow (Teklad Rodent Diet W-8604) and regular drinking water. Pregnant dams were given a breeder diet (Teklad S-2235 Mouse Breeder Sterilizable Diet 7004) through weaning. All experiments involving animal handling and care were approved by the University of California, Riverside IACUC.

For the previous study, one C line (Line 4) and one HR line (Line 7) were used because they represented extremes in body mass among their respective linetypes (Table 2 in Cadney et al. 2021b). As described, it was hypothesized that differences in dam body size would lead to differential cross-fostering effects, including wheel-running behavior of the pups at weaning. Thus, using lines with different body masses could serve as a positive control for offspring body masses at weaning. The wheel-running behavior of these lines was representative of their respective linetypes (Cadney et al. 2021b). The dams used in the previous study were typical of other line 4 and 7 breeders of the same generation in terms of both wheel-running and body mass (Cadney et al. 2021b).

### Experimental design

The experimental design has been previously published (Cadney et al. 2021b). Briefly, mice from generation 89 were sampled randomly to create a total of 60 C line 4 and 60 HR line 7 mating pairs, with the constraint that the sample size per foster group was equal (Table 1; Cadney et al. 2021b). A large number of pairings were made because only pups born on the same day could be used for fostering (Cadney et al. 2021b).

**Table 1:** Litter parameters for bisulfite sequencing analyses

Group	Litter	Female	Male	Pups
CC	5	6	5	11
CHR	5	6	6	12
HRC	5	6	6	12
HRHR	5	6	5	11
Total	20	24	22	46

Cortex and hippocampal tissue were used for DNA methylation analyses. All pups were maintained until sacrifice at 59 days of age

At birth, litters were standardized to eight pups to avoid litter effects. As sex could not be determined at birth, the litter sex ratio could not be controlled. Cross-fostering only occurred between litters born within 24 h of one another. During the 48 h after cross-fostering, fostered pups were checked regularly, and none were rejected by their foster mother.

**Fig. 1:**
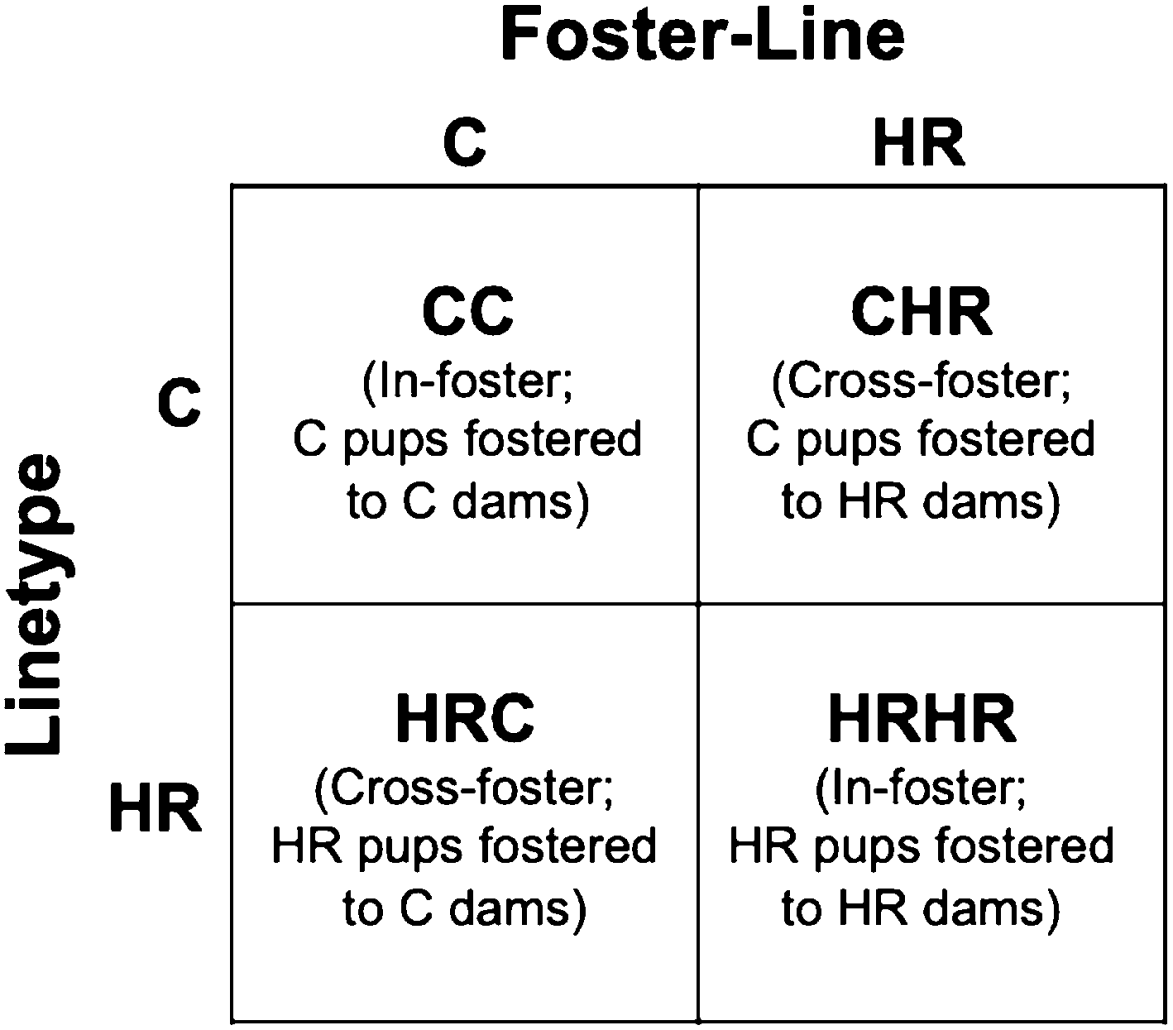
Experimental design. Four experimental groups were created by cross-fostering between families of C (line 4) and HR (line 7) mice

As births occurred, entire litters were fostered to another dam (no pup was returned to its biological mother). Thus, the previous study did not include a “control” group for the effects of fostering per se. This design was chosen to maximize the sample size in experimental groups sufficient to address their specific hypotheses (i.e., the effects of cross-fostering HR and C mice), given logistical constraints on the total sample size. In the current study, we wanted to determine whether rearing by an HR dam might be necessary for some proportion of high-running variance in DNA methylation profiles. This factorial experimental design produced four groups (Fig. 1 and Cadney et al. 2021b): C offspring raised by cross-fostered C dams (CC), C offspring raised by cross-fostered HR dams (CHR), HR offspring raised by cross-fostered C dams (HRC), and HR offspring raised by cross-fostered HR dams (HRHR). The number of male and female mice from each group used in this study is listed in Table 1.

Mice were removed from wheel access for one day, then sacrificed at seven weeks of age via decapitation without anesthesia. After whole brains were extracted, the hippocampus and cortex were dissected and snap-frozen on dry ice. Samples were stored at −80 °C until further sequencing steps were performed. Because of the reduced number of brain samples (N = 46), separate sex models could not be analyzed.

### In-silico assay design

Gene sequences were acquired from the Ensembl genome browser and annotated. The assay target sequences were then re-evaluated against the UCSC mouse GRCm38 genome browser for repeat sequences, including LINE, SINE, LTR elements, and other DNA repeats. Sequences containing repetitive elements, low sequence complexity, high thymidine content, and overall CpG density were excluded from the *in-silico* design process. Forty-two assays were designed to cover 293 CpG sites across 16 genes, and the percentage methylation of each CpG site was determined in each sample. A list of the genes, their respective coordinates, and the number of CpG sites analyzed in this study are provided in Table 2.

**Table 2:** Coordinates, genomic context, and number of CpG sites analyzed for 16 imprinted genes analyzed by bisulfite sequencing

Gene	Inheritance	GRCm38 Coordinates	From TSS	# Assays	Genomic Context	# CpG Sites
* Gnas*	Maternal	Chr2: 174,295,026–174,296,544	10,707 to 12,225	2	Intron 2	13
* Grb10 (Meg1)*	Maternal (Paternal in brain)	Chr11: 12,026,650	10,752	1	Intron 1	1
* H19*	Maternal	Chr7: 142,582,201–142,580,501	−4058 to −2358	3	5’ Upstream	22
* Ig-DMR*	Multiple	Chr12: 109,526,596–109,526,736	73,748 to 73,888	1	Ig-DMR	9
* Igf2*	Paternal (Maternal in brain)	Chr7: 142,669,731–142,669,634	−12,235 to −12,138	1	5’ Upstream	9
* Igf2r*	Maternal	Chr17: 12,742,702–12,742,174	26,963 to 27,491	2	Intron 2	18
* Impact*	Paternal	Chr18: 12,972,743–12,974,522	821 to 2600	2	Intron 1	14
* Kcnq1ot1*	Paternal	Chr7: 143,295,509–143,295,190	1041 to 1360	2	Exon 1	20
* Mest (Peg1)*	Paternal	Chr6: 30,736,749–30,737,751	−1301 to −299	4	5’ Upstream	28
		Chr6: 30,738,316–30,738,331	267 to 282	1	Exon 1	4
		Chr6: 30,738,352–30,739,354	303 to 1305	2	Intron 1	13
* Peg3*	Paternal	Chr7: 6,730,600–6,730,506	−179 to −85	1	5’ Upstream	6
		Chr7: 6,730,342–6,730,263	80 to 159	1	5’ UTR	9
		Chr7: 6,730,247–6,729,420	175 to 1002	3	Intron 1	24
* Plagl1*	Paternal	Chr10: 13,091,040–13,091,127	253 to 340	1	5’ Upstream	13
* Rasgrf1*	Paternal	Chr9: 89,870,292–89,879,770	−39,617 to −30,139	6	5’ Upstream	16
* Sgce*	Paternal	Chr6: 4,749,158–4,747,736	−1982 to −560	2	5’ Upstream	22
		Chr6: 4,747,042–4,747,006	135 to 171	1	Exon 1	4
		Chr6: 4,746,967–4,746,963	210 to 214	1	Intron 1	3
* Snrpn*	Paternal	Chr7: 60,005,229–60,004,853	−118 to 259	2	Intron 2	17
* Trappc9*	Multiple (Maternal in brain)	Chr15: 72,809,627–72,809,272	251,578 to 251,933	2	Intron 16	16
* Zdbf2*	Paternal	Chr2: 206,273,572–206,314,427	−10,439 to −9406	4	5’ Upstream	12
			Total number of CpG sites analyzed			293

Inheritance indicates parental expression. TSS indicates relative to the ATG transcription start codon. Negative sign indicates a location upstream of ATG; positive sign indicates a location downstream of ATG

### Bisulfite sequencing and data analysis

Bisulfite sequencing was performed on 92 samples (46 hippocampus and 46 cortex) by EpigenDx, Inc. (Hopkinton, MA). Tissue samples were digested using 500 μL of ZymoResearch M-digestion Buffer (Zymo, Irvine, CA) and 5–10 μL of protease K (20 mg/mL) with a final M-digestion concentration of 2X. The samples were incubated at 65 °C for a minimum of 2 h. 20 µL of the supernatant from the sample extracts were bisulfite modified using the ZymoResearch EZ-96 DNA Methylation-Direct Kit™ (cat# D5023) kit per the manufacturer’s protocol with minor modification. The bisulfite-modified DNA samples were eluted using M-elution buffer in 46 µL.

All bisulfite-modified DNA samples were amplified using separate multiplex or simplex PCRs with Qiagen (Gaithersburg, MD) HotStar Taq. All PCR products were verified and quantified using the QIAxcel Advanced System. Prior to library preparation, PCR products from the same sample were pooled and purified using QIAquick PCR Purification Kit columns (Qiagen).

Libraries were prepared using a custom Library Preparation method created by EpigenDx. Library molecules were then purified using Agencourt AMPure XP beads (Beckman Coulter) and quantified using the Qiagen QIAxcel Advanced System. Barcoded samples were then pooled in an equimolar fashion before template preparation and enrichment were performed on the Ion Chef™ system (Thermo Fisher) using Ion 520™ & Ion 530™ ExT Chef reagents. Following this, enriched, template-positive library molecules were sequenced on the Ion S5™ sequencer using an Ion 530™ sequencing chip (Thermo Fisher).

FASTQ files from the Ion Torrent S5 server were aligned to the local reference database using open-source Bismark Bisulfite Read Mapper with the Bowtie2 alignment algorithm (https://www.bioinformatics.babraham.ac.uk/projects/bismark/; Krueger and Andrews 2011). Methylation levels were calculated in Bismark by dividing the number of methylated reads by the total number of reads, considering all CpG sites covered by a minimum of 30 total reads. CpG sites with fewer than 30 reads were excluded from analyses.

### Statistical analysis

Data were analyzed as mixed models in SAS 9.1.3 (SAS Institute, Cary, NC) Procedure Mixed, with REML estimation and Type III Tests of Fixed Effects. Line (selected line 7 vs. non-selected line 4), foster-line, and sex were fixed effects, while dam ID (n = 28) was a random effect nested within linetype × foster-line. Separate mixed models were created to analyze the percent methylation levels for CpG sites across the entire genomic region for each gene (Supplementary Table 1) as well as the percent methylation levels for CpG sites within distinct genomic regions (i.e., introns, exons, 5’ upstream regions, and 5’ untranslated regions; Supplementary Table 2).

In all tables, we present least squares means (L.S. mean) and standard errors (S.E.) for each imprinted gene for both brain regions (cortex and hippocampus) and the results of each F-statistic. Supplementary Tables 1 and 2 also present the p-values for the differences in the L.S. means between the in-fostered and cross-fostered groups (i.e., the effect of cross-fostering between the HR and C lines by sex). Supplemental material can also be referenced for main effects, as well as interactions (line × foster-line, line × sex, foster-line × sex, line × foster-line × sex).

In all analyses, outliers were iteratively removed when the standardized residuals exceeded ~ 3. Statistical significance was set at p < 0.05. Effect sizes, presented as Hedges’ g values, were also calculated for group comparisons (CC vs. HRHR, CC vs. CHR, and HRHR vs. HRC; Tables 3 and 4 and Supplemental Tables 3 and 4). Hedges’ g values greater than  + 0.8 and less than − 0.8 were viewed as a large effect size, while values between + 0.8 and + 0.5 and between − 0.8 and − 0.5 were considered to be a medium effect size.

**Table 3:**
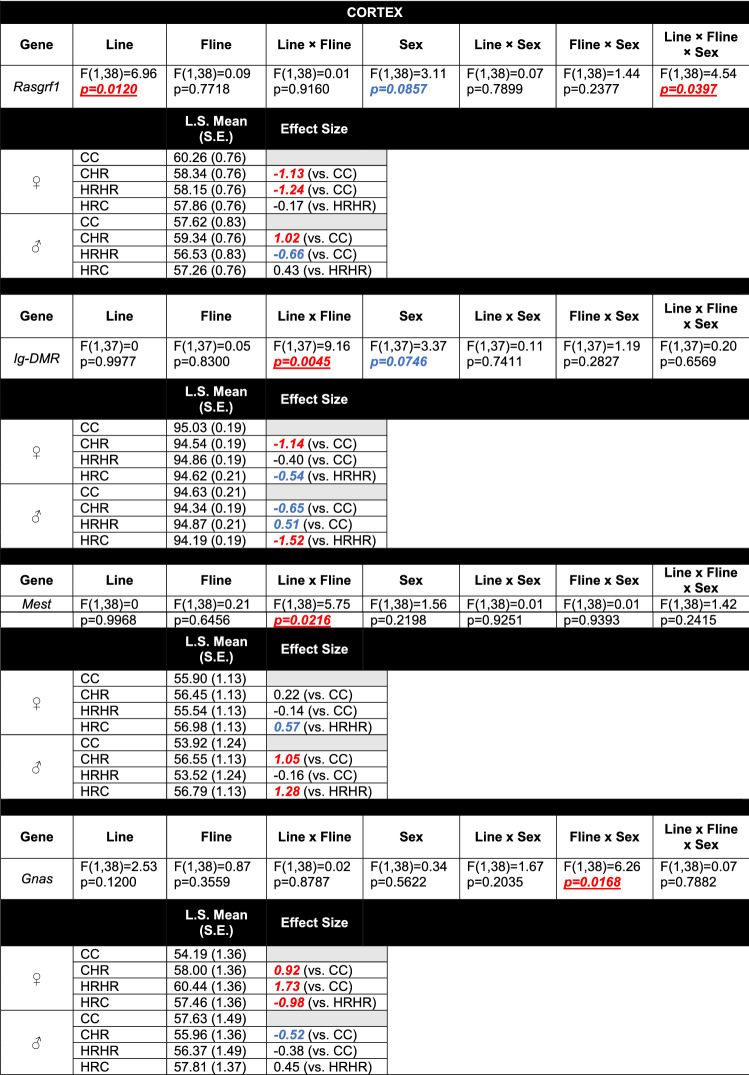

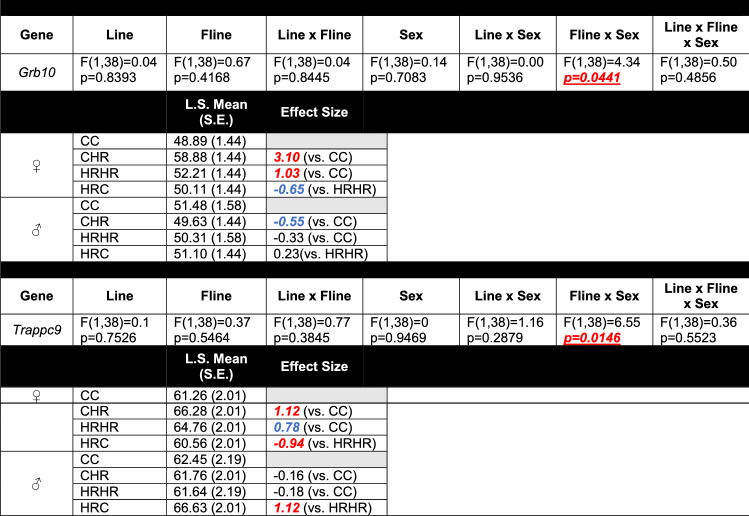
Type 3 tests of fixed effects for genes in the cortex with at least one significant main effect and/or interaction (Color table online)

Line, foster-line, sex, line × fline, line × sex, fline × sex, line × fline × sex were included as terms in all models. Separate models were run for each gene. F-statistic and associated p-values for each gene are reported. Hedges’ g value from select comparisons is also reported (CC vs. HRHR; CC vs. CHR; HRHR vs. HRC). Values ± 0.8 or greater (indicated in red) were viewed as large effect sizes. Values between ± 0.5 and ± 0.8 (indicated in blue) were considered as a medium effect size

**Table 4:**
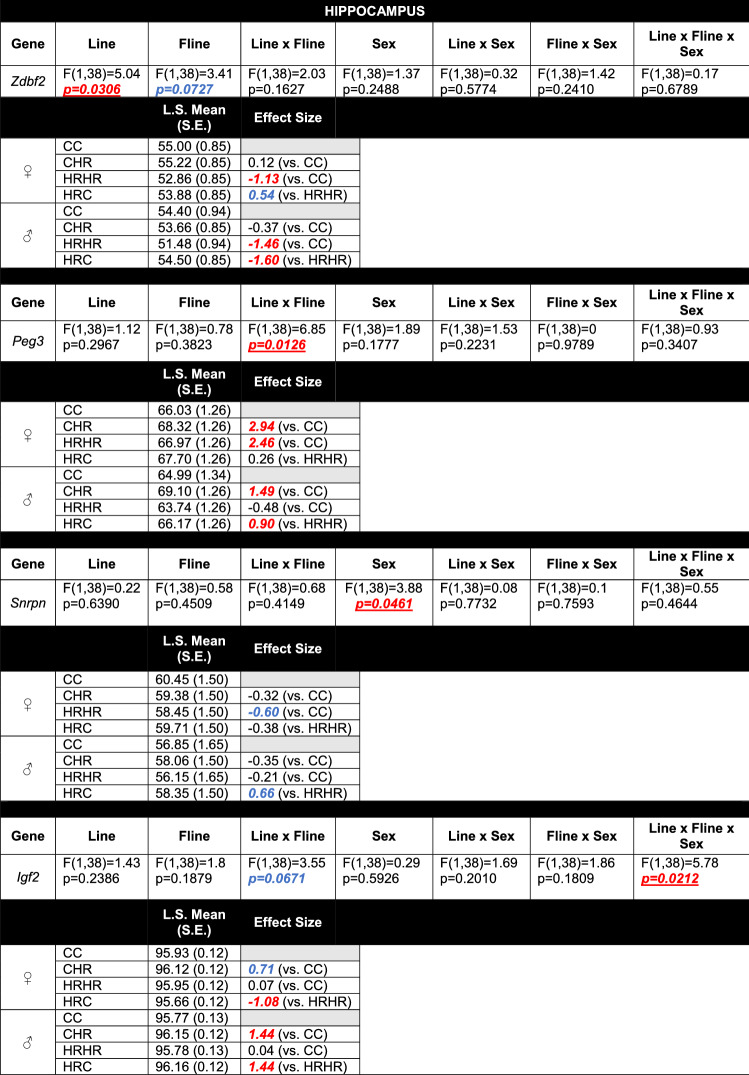

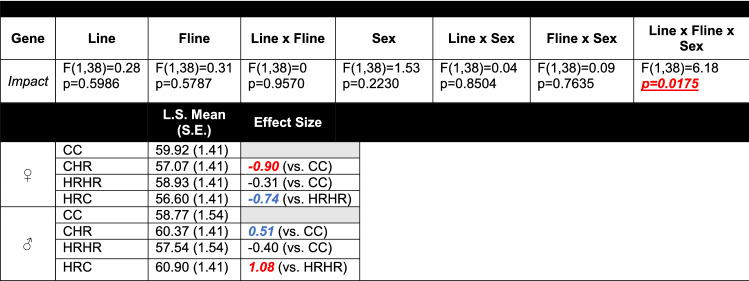
Type 3 tests of fixed effects for genes in the hippocampus with at least one significant main effect and/or interaction (Color table online)

Line, foster-line, sex, line × fline, line × sex, fline × sex, line × fline × sex were included as terms in all models. Separate models were run for each gene. F-statistic and associated p-values for each gene are reported. Hedges’ g value from select comparisons is also reported (CC vs. HRHR; CC vs. CHR; HRHR vs. HRC). Values ± 0.8 or greater (indicated in red) were viewed as large effect sizes. Values between ± 0.5 and ± 0.8 (indicated in blue) were considered a medium effect size

## Results

To address whether mice from a line genetically selected for elevated voluntary physical activity have altered DNA methylation profiles of imprinted genes compared with non-selected C mice, we investigated the methylation profile of C offspring that were cross-fostered at birth and reared by C dams and HR offspring that were cross-fostered at birth and raised by HR dams. We also investigated the methylation profiles of C offspring cross-fostered at birth and reared by HR dams and HR offspring cross-fostered at birth and raised by C dams to investigate additional early-life programming effects (i.e., maternal effects) on genomic imprinting (Fig. 1). This factorial experimental design allowed us to examine the genomic and non-genomic contributions to elevated physical activity. Because the expression of imprinted genes varies with tissue type and male and female mice differ in wheel-running behaviors, we examined the DNA methylation profiles in the hippocampus and cortex of both sexes. The percent methylation of 16 imprinted genes totaling to 293 CpG sites were analyzed by bisulfite sequencing and are reported in Supplemental Tables 1 and 2. The F-statistic and accompanying p-values for those that had p < 0.05 for a main effect or interaction are further reported in Tables 3 and 4 for the cortex and hippocampus, respectively, as well as Supplemental Tables 3 and 4. Hedges’ g value of effect sizes between key groups (CC vs. HRHR, CC vs. CHR, and HRHR vs. HRC) for these genes are also reported.

### Individual main effects of line and maternal upbringing on DNA methylation

When analyzed across the entire genomic region, our statistical model revealed several significant individual main effects of line (physical activity) and foster-line (maternal upbringing) on the DNA methylation profiles of the 16 imprinted genes analyzed in this study (Supplemental Table 1). A significant main effect of line for *Rasgrf1* in the cortex (p = 0.0120; Table 3) and *Zdbf2* in the hippocampus (p = 0.0306; Table 4) was observed, as well as additional trends towards statistical significance (p < 0.10) for foster-line for *Zdbf2*, (p = 0.0727; Table 4) in the hippocampus.

When the CpG sites were analyzed within distinct genomic regions such as introns, exons, and promoter regions, no significant individual main effects of line and foster-line on the DNA methylation of the 16 imprinted genes were revealed (Supplemental Tables 3 and 4). These results suggest that differences in genomic regions do not significantly influence the overall methylation profile of any of the imprinted genes analyzed.

### Interaction of line and maternal upbringing on DNA methylation

When assessed for line and foster-line interaction across the entire gene, there were significant main effects for *Ig-DMR* (p = 0.0045) and *Mest* (p = 0.0216) in the cortex (Table 3). The hippocampus had a significant main effect for line and foster-line for *Peg3* (p = 0.0126; Table 4). When the CpG sites were analyzed by genomic context, there were additional line × foster-line interactions (Supplemental Tables 3 and 4). In the cortex, there was a significant line × foster-line interaction for the exon (p = 0.0063) and promoter (p = 0.0483) regions of *Mest* (Supplemental Tables 3). In the hippocampus, there was a significant line × foster-line interaction for the intron region of *Peg3* (p = 0.0062; Supplemental Tables 4).

### Influence of sex and the interaction of sex, line, and maternal upbringing on DNA methylation

When analyzed across the entire genomic region, sex produced an individual main effect on *Snrpn* in the hippocampus (p = 0.0461; Table 4). A trend for a significant main effect of sex for *Rasgrf1* (p = 0.0857) and *Ig-DMR* (p = 0.0746) in the cortex (Table 3) and *Igf2* (p = 0.0671) in the hippocampus (Table 4) were also observed. When assessed for a three-way line, foster-line, and sex interaction, there were significant main effects for *Rasgrf1* (p = 0.0397) in the cortex (Table 3) and *Igf2* (p = 0.0212) and *Impact* (p = 0.0175) in the hippocampus (Table 4). The *Sgce* intron region within the hippocampus also revealed an additional three-way interaction (p = 0.0168; Supplemental Tables 4).

Analyses of effect size between CC vs. CHR, CC vs. HRHR, and HRHR vs. HRC groups revealed large effect sizes with Hedges’ g values of greater than + 0.8 and/or less than − 0.8 for *Rasgrf1*, *Ig-DMR*, *Mest*, *Gnas*, *Grb10*, and *Trappc9* in the cortex (Table 3 and Supplemental Tables 3) and *Zdbf2*, *Peg3, Igf2,* and *Impact* in the hippocampus (Table 4 and Supplemental Tables 4), indicating notable effects of line and foster-line, despite non-significant line × foster-line interactions for these genes.

## Discussion

The DNA methylation status of genes is an attractive conduit for early-life effects that can persist into adulthood. Moreover, alterations in DNA methylation can interact with other early-life influences and amplify across successive generations (Yin et al. 2013; Short et al. 2017; Yeshurun et al. 2017; McGreevy et al. 2019). Here we analyzed DNA methylation patterns for 16 imprinted genes in a genetically selected line of mice (HR) and a non-selected line (C) whose offspring were cross-fostered in a full factorial experimental design. Our results shed light on non-genomic contributions to elevated physical activity. The results from our statistical model, which accounted for the influence of sex, line, and foster-line, showed that lines differed in DNA methylation patterns of *Rasgrf1* in the cortex (Table 3) and *Zdbf2* in the hippocampus (Table 4). These results support the hypothesis that the genetic inheritance of elevated physical activity can potentially lead to altered expression of imprinted genes in the brain. Furthermore, early-life effects through cross-fostering modified the methylation status of other imprinted genes involved in fetal growth and energy metabolism, including *Ig-DMR*, *Mest*, and *Peg3*. Finally, the sexes differed in DNA methylation for the paternally expressed genes *Snrpn*, *Rasgrf1*, *Igf2*, and *Impact*.

Below, we first discuss the significance of the imprinted genes, *Rasgrf1* and *Zdbf2*, given that the methylation patterns of these genes were significantly affected by wheel-running. We then discuss the methylation status of additional imprinted genes modified with cross-fostering and by sex and the implications of these genes on neurodevelopment.

### The HR line and genomic imprinting of *Rasgrf1* and *Zdbf2* in the brain

Our most notable finding was alterations in DNA methylation in the paternally imprinted genes, *Rasgrf1* and *Zdbf2,* in the cortex and hippocampus, respectively, in the HR line (Tables 3 and 4). In mice, *Rasgrf1* (Ras protein-specific guanine nucleotide releasing factor 1) is a paternally methylated and paternally expressed imprinted gene located on chromosome 9 (Plass et al. 1996; Yoon et al. 2002; Dockery et al. 2009). All 16 CpG sites assayed for this gene were located approximately 30 kb to 40 kb upstream of the transcription start site, where the *Rasgrf1* differentially methylated region (DMR) is located (Shibata et al. 1998; Dockery et al. 2009). The methylation status of the *Rasgrf1* DMR directly influences *Rasgrf1* expression in the brain. The *Rasgrf1* DMR contains CTCF binding sites, which have been shown to function as enhancer blockers at maternally imprinted loci, including *H19*, *Igf2,* and *KvDMR1* (Bell and Felsenfeld 2000; Hark et al. 2000; Yoon et al. 2005; Holmes et al. 2006; Fitzpatrick et al. 2007). CTCF binds to the unmethylated *Rasgrf1* DMR, which functions in *cis* to silence the maternal allele (Yoon et al. 2005; Fitzpatrick et al. 2007). Experimental conditions that prevent methylation of the paternal DMR result in silencing the normally expressed paternal allele in the brain, consistent with the model that the unmethylated DMR binds CTCF, blocking expression of the *Rasgrf1* allele in *cis* (Yoon et al. 2002, 2005). Adding an enhancer between the DMR and *Rasgrf1* overrides the enhancer-blocking activity of the CTCF-bound DMR, implicating enhancers located upstream of the DMR in the regulation of *Rasgrf1* in the brain (Yoon et al. 2005; Holmes et al. 2006).

*Rasgrf1* is detected mainly in the brain (Plass et al. 1996; Yoon et al. 2002; Holmes et al. 2006; Fernandez-Medarde and Santos 2011), and its expression changes from exclusively paternal before weaning to preferentially paternal after weaning (Drake et al. 2009). Mice with *Rasgrf1* paternal deletions are normal at birth but display retarded growth by weaning (Itier et al. 1998), and loss of *Rasgrf1* imprinting increases growth and weight after birth (Drake et al. 2009). Synchronization of growth in the developing brain relies on the secretion of growth hormone (GH)-releasing hormone and somatostatin, which stimulate and inhibit GH and IGF1 secretion, respectively. Levels of both GH and IGF1 decrease or increase when *Rasgrf1* is deleted or overexpressed, respectively.

*Rasgrf1* also mediates oxidative stress and inflammatory responses and can be upregulated with chronic exercise (Table 3; Moreland et al. 2020) and stress (Cheng et al. 2020). *Rasgrf1*-deficient mice display lower levels of reactive oxygen species, protection against oxidative stress, reduced body size, and improved motor coordination with age (Borras et al. 2011). It is possible that the alteration of *Rasgrf1* methylation in HR mice results in a shift in oxidant and anti-oxidant factors and could contribute to the neurobiological alterations observed in these mice. Supporting this, *Rasgrf1* is expressed in the postsynaptic densities of neurons and is an intrinsic mediator of brain derived neurotrophic factor-induced small GTPase R-Ras activation, R-Ras-mediated axonal morphological regulation (Umeda et al. 2019), and is involved in memory formation (Brambilla et al. 1997).

We also observed a significant main effect of selective breeding for elevated physical activity for 12 CpG sites located 10 kb upstream of the *Zdbf2* transcription start site (Table 4). The *Zdbf2* (DBF-type zinc finger-containing protein 2) gene encodes for a protein containing DBF4-type zinc finger domains but has no known function. Like *Rasgrf1*, *Zdbf2* is also paternally methylated and paternally expressed (Kobayashi et al. 2009) and controls the paternal-specific expression of *Zdbf2* isoforms during early development (Duffie et al. 2014). The *Zdbf2* locus is paternally imprinted, with three intergenic paternally methylated DMRs located between 8.5 kb and 16 kb upstream of the *Zdbf2* transcription start site. However, additional work suggests that the *Zdbf2* DMR locus may also be under transient maternal imprinting control during specific developmental periods (Kobayashi et al. 2012; Proudhon et al. 2012; Duffie et al. 2014), likely due to multiple promoters (Duffie et al. 2014). Because the function of *Zdbf2* is unknown, it is difficult to estimate the functional consequences of altered *Zdbf2* methylation in the hippocampus of our HR mouse model. However, studies have shown that aberrant hypermethylation of the maternal *Zdbf*2-DMR has been observed in some patients with Beckwith-Wiedemann Syndrome (Maeda et al. 2014), an imprinting disorder characterized by body overgrowth and enlargement of internal organs. Investigating the functions of the *Zdbf2* gene in exercise behavior may provide further information about how *Zdbf2* expression may control fetal growth and metabolism.

Because the two genes that were significantly altered by selective breeding are paternally methylated and paternally expressed genes, our results support a hypothesis that paternal imprinting of genes in the brain may be a conduit through which exercise behavior influences nervous system development and function (Radford et al. 2011; Li et al. 2013; Baker et al. 2015; Eclarinal et al. 2016; Garland et al. 2017). In addition, given the well-characterized changes in allele frequencies for genes associated with elevated levels of exercise (Xu and Garland 2017; Hillis et al. 2020), it is also plausible that genetic and epigenetic factors interact with each other to modify DMR methylation profiles, as has been noted for human *IGF2R* (Xu et al. 1997; Sandovici et al. 2003) and *IGF2* (Sandovici et al. 2003) genes. This possibility remains to be tested in future studies.

### Influence of maternal upbringing on genomic imprinting in the brain

The early family life environment shapes human neurodevelopment and behavioral outcomes well into adulthood (Heim et al. 2002; Apter-Levy et al. 2013). In rodents, naturally occurring variations in maternal care shape the offspring’s neuroendocrine stress reactivity, anxiety, and depression-like behavior (Meaney 2001; Schroeder and Weller 2010; Kessler et al. 2011; Cohen et al. 2015). Dams exhibiting different social behaviors also demonstrate variations in maternal care (Clinton et al. 2007, 2010), and cross-fostering their offspring shifts their adult phenotype, leading to greater social interaction and reduced anxiety-like anxiety behavior. Anxiety-like behavior effects are accompanied by gene expression changes in the amygdala that emerge in early postnatal life and persist through adulthood, revealing how an early-life manipulation such as cross-fostering can change brain development and ultimately impact adult behavior.

Variations in maternal care can also alter the expression of imprinted genes. It has been suggested that paternally expressed imprinted genes increase offspring size and survival. Mutations affecting the paternally expressed *Mest* (Lefebvre et al. 1998) and *Peg3* (Li et al. 1999; Curley et al. 2004) genes result in a striking lack of maternal care, supporting the notion that genomic imprinting is associated with the evolution of social behaviors. Targeted mutation of paternally expressed imprinted genes influences several aspects of fetal and postnatal development. Mutation of *Peg3* in offspring reared with wild-type mothers, and vice versa, significantly increases offspring mortality, with the combined mutation in mother and offspring exhibiting near 100% lethality (Curley et al. 2004). Moreover, *Peg3* mutant mothers exhibit decreased nurturing behaviors, including nest-building, crouching, and pup retrieval, as well as reduced numbers of oxytocin-producing neurons in the hypothalamus (Li et al. 1999). Inactivation of *Mest* in embryonic cells also resulted in abnormal maternal behavior directly impacting the feeding behavior of newborns (Lefebvre et al. 1998). These data suggest that paternally imprinted genes are critical for maternal care. Consistent with these data, we observed alterations in the methylation status of four paternally expressed genes, *Mest*, *Peg3*, *Ig-DMR*, and *Impact*, in cross-fostered HR mice (Tables 3 and 4). These genes have been shown to promote the growth of body size but inhibit the growth of the brain (Keverne et al*.* 1996, Bouschet et al. 2017). Given that these genes play critical roles in fetal growth and metabolism, these data support the hypothesis that DNA methylation may be a mechanism to explain the persistence of developmental programming influences on exercise behavior.

Although the observed changes in DNA methylation levels in the current study may directly influence wheel-running behavior, this hypothesis is challenging to assess. In Cadney et al. (2021b), HR pups raised by HR dams weighed less than C pups raised by C dams and continued to have reduced body masses as adults. As expected, adult HR mice also ran approximately threefold more than their C counterparts, and females ran more than males. However, cross-fostering had no statistical consequence on any aspect of wheel-running behavior, including running distance, duration, or maximum speed. In addition, with body mass as a covariate, HR mice had higher VO_2max_ than C mice, and males had higher VO_2max_ than females, but cross-fostering had no effect. Related to the brain, female HR pups raised by C dams had significantly larger brains than female HR pups raised by HR dams (Cadney et al. 2021b). Because cross-fostering had no effect on adult exercise levels, future studies focusing on the methylation profiles of other tissues relevant to exercise physiology (e.g., skeletal muscle, heart, liver) are not likely to reveal important effects of cross-fostering on voluntary exercise behavior in these lines of mice. However, potential epigenetic effects induced by other early-life environmental factors, such as altered diet and/or exercise (Li et al. 2013; Desai et al. 2014a, 2014b; Meek et al. 2014; Acosta et al. 2015; Hiramatsu et al. 2017; Cadney et al. 2021a, 2022), remain an important area of focus for additional study.

The methylation profiles at most of the imprinted gene DMRs analyzed in the current study had a baseline level of methylation close to the theoretical 50% expected for loci whose methylation status is differentially established on parental alleles. However, two genes, *Igf2* and *Ig-DMR*, had almost 100% methylation in the cortex and hippocampus (Supplemental Table 1 and Tables 3 and 4). These unexpected findings prompted us to consider the identity and nature of such early-life effects that may be capable of shifting gene methylation profiles. Many developmental exposures have been demonstrated to shift the DMR methylation profile for *Igf2*, including maternal cigarette smoking (Murphy et al. 2012a), maternal stress (Vangeel et al. 2015), anti-depressant medications taken during pregnancy (Soubry et al. 2011), prenatal nutrition (Kovacheva et al. 2007; Heijmans et al. 2008; Tobi et al. 2009; Hoyo et al. 2011), postnatal nutrition (Waterland et al. 2006; Hoyo et al. 2011), and chemical exposure (Robles-Matos et al. 2021). Such deviations in DMR methylation are likely functionally significant, given that imprinted genes are critical for cellular differentiation (Zhang et al. 1997), prenatal and postnatal growth (Bouschet et al. 2017), neurobiological function (Isles and Wilkinson 2000; Perez et al. 2016; Huang et al. 2017; Kravitz and Gregg 2019), and maternal behaviors (Lefebvre et al. 1998; Li et al. 1999).

The imprinting of *Igf2* (Gregg et al. 2010; Harper et al. 2014; Ye et al. 2015), *Dlk1* (Croteau et al. 2005), *Grb10* (Monk et al. 2009; Garfield et al. 2011), and *Peg1/Mest* (Shi et al. 2004) DMRs in the brain may also not be as strictly maintained in other tissues. Such epigenetic heterogeneity may be partially explained by the relaxation of epigenetic control for genes whose monoallelic expression is not essential for brain function. However, this is an unlikely interpretation because *Igf2* and *Ig-DMR* are critical for neurodevelopment. An alternative and more probable interpretation is that variation between monoallelic and biallelic expression of imprinted genes in the brain may be directly related to brain function and may reflect a response to changes in environmental cues, such as the maternal environment. In one study, *Igf2* was shown to be abundantly expressed in the hippocampus and prefrontal cortex of adult rats and, in contrast to peripheral tissues, was maternally expressed (Ye et al. 2015). Given the established role of imprinted genes in maternal behavior and neurobiological processes (Lefebvre et al. 1998; Li et al. 1999), it is reasonable to conjecture that increased plasticity of imprinting in the brain may contribute to complex and heritable behavioral phenotypes, including exercise behavior. Additional insights are further discussed below.

### Sexual dimorphism in parental imprinting in the brain

Earlier studies show that when provided access to a running wheel, females run significantly more, for more extended periods, and at higher average speeds than males (Swallow et al. 1998). This was also seen when offspring of HR females ran significantly longer distances, spent more time running, and ran at higher maximum speeds than the offspring of HR males (Kelly et al. 2010). As a result, females were also smaller and had lower percentage of body fat (Swallow et al. 1999) and generally regulated energy balance more precisely than males (Swallow et al. 1999). In the current study, sex further modified DMR methylation levels for *Rasgrf1* in the cortex (Table 3) and *Snrpn*, *Igf2*, and *Impact* in the hippocampus (Table 4), all of which are paternally imprinted. These findings were expected, given the numerous sex-specific effects observed in the previous cross-fostering study, with cross-fostering increasing brain mass of female, but not male, HR mice (Cadney et al. 2021b).

Several reasons could account for sex differences in DNA methylation. First, the timing and duration of methylation marks are different in the male versus the female germlines (Bourc'his and Proudhon 2008). At the time of fertilization, paternal imprinting control regions are methylated in spermatozoa versus unmethylated in the oocyte. In contrast, maternal imprinting control regions are methylated in the oocyte but not in spermatozoa. Second, the developmental stage in which gametic methylation imprints are acquired is also sex-specific, with de novo methylation being initiated earlier in the male germline compared to the female germline. Methylation is acquired very early during spermatogenesis, in precursors of the self-renewing spermatogonial stem cells. Because of this, paternally methylated CpGs endure more replication cycles in spermatogonial stem cells and proliferating spermatogonia and persist several weeks to several years before the production of mature spermatozoa (Eichenlaub-Ritter et al. 2007). On the contrary, maternal methylation patterns are established just before ovulation. The longer duration of paternal methylation patterns and the higher number of replication cycles during gametogenesis may favor the erasure of paternal ICRs, while the shorter longevity of methylation patterns in the female germline may be better conserved. Third, while the total number of paternally and maternally imprinted genes is approximately even, most of the primary methylation marks acquired in the germline are of maternal origin. More than 15 imprinted clusters are dependent on ICRs harboring maternal germline methylation, while only three imprinted loci are controlled by paternal germinal marks: *H19/Igf2*, *Gtl2/Dlk1,* and *A19/Rasgrf1* (Reik and Walter 2001). The rest of the paternally imprinted genes are controlled through the production in *cis* of a non-coding RNA, silencing itself on the maternal allele by DNA methylation inherited from the oocyte. Lastly, the maternal genome is required for the development of the embryo, while the paternal genome promotes the development of extraembryonic structures such as the placenta (Barton et al. 1984; McGrath and Solter 1984). However, it is difficult to address the specific influence of maternal and paternal germline imprints on the neurodevelopment of the offspring. Regardless, it is reasonable to deduce that sex-specific observations such as those found in our study may be informative when designing future studies to evaluate links between parent-of-origin genes and sexually dimorphic effects on wheel-running behavior.

### Limitations and concluding remarks

The current study provides new insights into potential epigenetic mechanisms underlying the developmental origins of exercise and physical activity. However, some limitations will need to be addressed in future studies. First, the cross-fostering experimental design did not include non-fostering controls such that C offspring were reared by their biological C dam, and HR offspring were reared by their biological HR dam. This limitation was intentional, as the inclusion of such controls was not necessarily relevant to the experimental aims of the previous study. Additionally, resource constraints and early pandemic restrictions on in-person research were also concerns. Consequently, the current experimental design was limited to 20 litters. Second, the DNA in this study was extracted from hippocampal and cortical tissue made up of a heterogeneous collection of cells. DNA methylation can vary by cell type, so this must be carefully considered in future studies (Davies et al. 2005; Kravitz and Gregg 2019). Cell-type-specific imprinting effects are especially intriguing because they could serve as potential markers for novel subpopulations of brain cells controlling a particular neural circuit or behavior pattern. Third, we did not assess the ability of DNA methylation status to modulate gene expression; therefore, gene expression studies are needed to assess gene activity. Lastly, bisulfite sequencing does not differentiate between 5-methylcytosine and 5-hydroxymethylcytosine. This is critical because 5-hydroxymethylcytosine is enriched in the brain and is regulated during development (Kinney et al. 2011; Sherwani and Khan 2015). In the future, it will be essential to determine whether alterations in DNA methylation associated with physical activity and other early-life programming factors are mediated by enzymes that add not only 5-methylcytosines, but also 5-hydroxymethylcytosines.

Although we have observed line and sex-specific changes in DNA methylation patterns of several imprinted genes, it is prudent to question the relevance and potential functional consequences of small percentage changes in DNA methylation like those measured in this study (Breton et al. 2017). Indeed, several studies have found that small changes in CpG methylation are correlated with differential gene expression (Murphy et al. 2012b; Kile et al. 2013; Argos et al. 2015; Maccani et al. 2015; Montrose et al. 2017). Therefore, it is possible that even small shifts in DNA methylation that produce large effect sizes can directly impact the transcription and expression of these genes.

Because epigenetic marks can “drift” over time, it will also be critical to assess offspring later in life (Kochmanski et al. 2017) to determine whether DNA methylation changes are maintained, possibly amplify with age, and/or transmitted across generations. The expression of imprinted genes can also be regulated by additional epigenetic mechanisms, such as histone modifications, alternative promoter usage, and post-transcriptional processes (Jouvenot et al. 1999; Landers et al. 2004; Kim et al. 2018). Investigating the contribution of additional epigenetic mechanisms in the regulation of wheel-running behavior is also warranted.

In conclusion, using a cross-fostering paradigm to investigate non-genomic contributions to increased physical activity, we show that a line of mice selectively bred for high voluntary wheel-running behavior has stable alterations in DNA methylation levels of imprinted genes with known functions for fetal growth, development, and metabolism. These differential methylation patterns were also shaped by maternal upbringing and sex, supporting the hypothesis that genomic imprinting in the brain can contribute to complex and heritable behavioral phenotypes, and is further influenced by developmental programming factors during early life.

## Supplementary information

Below is the link to the electronic supplementary material.

Supplementary file1 (XLSX 309 KB)

Supplementary file2 (XLSX 195 KB)

Supplementary file3 (DOCX 27 KB)

Supplementary file4 (DOCX 16 KB)

## Data Availability

All data generated and analyzed during this study are included in this published article and its supplementary information files.

## References

[CR1] Aasdahl L, Nilsen TIL, Meisingset I, Nordstoga AL, Evensen KAI, Paulsen J, Mork PJ, Skarpsno ES (2021). Genetic variants related to physical activity or sedentary behaviour: a systematic review. Int J Behav Nutr Phys Act.

[CR2] Acosta W, Meek TH, Schutz H, Dlugosz EM, Vu KT, Garland T (2015). Effects of early-onset voluntary exercise on adult physical activity and associated phenotypes in mice. Physiol Behav.

[CR3] Albrecht U, Sutcliffe JS, Cattanach BM, Beechey CV, Armstrong D, Eichele G, Beaudet AL (1997). Imprinted expression of the murine angelman syndrome gene, Ube3a, in hippocampal and purkinje neurons. Nat Genet.

[CR4] Apter-Levy Y, Feldman M, Vakart A, Ebstein RP, Feldman R (2013). Impact of maternal depression across the first 6 years of life on the child’s mental health, social engagement, and empathy: the moderating role of oxytocin. Am J Psychiatry.

[CR5] Argos M, Chen L, Jasmine F, Tong L, Pierce BL, Roy S, Paul-Brutus R, Gamble MV, Harper KN, Parvez F, Rahman M, Rakibuz-Zaman M, Slavkovich V, Baron JA, Graziano JH, Kibriya MG, Ahsan H (2015). Gene-specific differential DNA methylation and chronic arsenic exposure in an epigenome-wide association study of adults in Bangladesh. Environ Health Perspect.

[CR6] Asmus F, Zimprich A, Du Montcel ST, Kabus C, Deuschl G, Kupsch A, Ziemann U, Castro M, Kühn AA, Strom TM, Vidailhet M, Bhatia KP, Durr A, Wood NW, Brice A, Gasser T (2002). Myoclonus-dystonia syndrome: epsilon-sarcoglycan mutations and phenotype. Ann Neurol.

[CR7] Azzi S, Abi Habib W, Netchine I (2014). Beckwith-wiedemann and russell-silver syndromes: from new molecular insights to the comprehension of imprinting regulation. Curr Opin Endocrinol Diabetes Obes.

[CR8] Babak T, Deveale B, Armour C, Raymond C, Cleary MA, van der Kooy D, Johnson JM, Lim LP (2008). Global survey of genomic imprinting by transcriptome sequencing. Curr Biol.

[CR9] Babak T, DeVeale B, Tsang EK, Zhou Y, Li X, Smith KS, Kukurba KR, Zhang R, Li JB, van der Kooy D, Montgomery SB, Fraser HB (2015). Genetic conflict reflected in tissue-specific maps of genomic imprinting in human and mouse. Nat Genet.

[CR10] Baker MS, Li G, Kohorst JJ, Waterland RA (2015). Fetal growth restriction promotes physical inactivity and obesity in female mice. Int J Obes (lond).

[CR11] Barlow DP, Bartolomei MS (2014). Genomic imprinting in mammals. Cold Spring Harb Perspect Biol.

[CR12] Bartolomei MS, Ferguson-Smith AC (2011). Mammalian genomic imprinting. Cold Spring Harb Perspect Biol.

[CR13] Barton SC, Surani MA, Norris ML (1984). Role of paternal and maternal genomes in mouse development. Nature.

[CR14] Bell AC, Felsenfeld G (2000). Methylation of a CTCF-dependent boundary controls imprinted expression of the Igf2 gene. Nature.

[CR15] Borras C, Monleon D, Lopez-Grueso R, Gambini J, Orlando L, Pallardo FV, Santos E, Vina J, Font de Mora J (2011). RasGrf1 deficiency delays aging in mice. Aging (albany NY).

[CR16] Bourc’his D, Proudhon C (2008). Sexual dimorphism in parental imprint ontogeny and contribution to embryonic development. Mol Cell Endocrinol.

[CR17] Bouschet T, Dubois E, Reynes C, Kota SK, Rialle S, Maupetit-Mehouas S, Pezet M, Le Digarcher A, Nidelet S, Demolombe V, Cavelier P, Meusnier C, Maurizy C, Sabatier R, Feil R, Arnaud P, Journot L, Varrault A (2017). In vitro corticogenesis from embryonic stem cells recapitulates the in vivo epigenetic control of imprinted gene expression. Cereb Cortex.

[CR18] Brambilla R, Gnesutta N, Minichiello L, White G, Roylance AJ, Herron CE, Ramsey M, Wolfer DP, Cestari V, Rossi-Arnaud C, Grant SG, Chapman PF, Lipp HP, Sturani E, Klein R (1997). A role for the Ras signalling pathway in synaptic transmission and long-term memory. Nature.

[CR19] Breton CV, Marsit CJ, Faustman E, Nadeau K, Goodrich JM, Dolinoy DC, Herbstman J, Holland N, LaSalle JM, Schmidt R, Yousefi P, Perera F, Joubert BR, Wiemels J, Taylor M, Yang IV, Chen R, Hew KM, Freeland DM, Miller R, Murphy SK (2017). Small-magnitude effect sizes in epigenetic end points are important in children’s environmental health studies: the children’s environmental health and disease prevention research center’s epigenetics working group. Environ Health Perspect.

[CR20] Cadney MD, Hiramatsu L, Thompson Z, Zhao M, Kay JC, Singleton JM, Albuquerque RL, Schmill MP, Saltzman W, Garland T (2021). Effects of early-life exposure to Western diet and voluntary exercise on adult activity levels, exercise physiology, and associated traits in selectively bred high runner mice. Physiol Behav.

[CR21] Cadney MD, Schwartz NE, McNamara MP, Schmill MP, Castro AA, Hillis DA, Garland T (2021). Cross-fostering selectively bred high runner mice affects adult body mass but not voluntary exercise. Physiol Behav.

[CR22] Cadney MD, Albuquerque RL, Schwartz NL, McNamara MP, Castro AA, Schmill MP, Garland T (2022). Effects of early-life exposure to fructose and voluntary exercise on adult activity levels, body composition, exercise physiology, and associated traits in mice. JDOHaD in Revision.

[CR23] Caetano-Anolles K, Rhodes JS, Garland T, Perez SD, Hernandez AG, Southey BR, Rodriguez-Zas SL (2016). Cerebellum transcriptome of mice bred for high voluntary activity offers insights into locomotor control and reward-dependent behaviors. PLoS ONE.

[CR24] Cassidy SB, Dykens E, Williams CA (2000). Prader-Willi and angelman syndromes: sister imprinted disorders. Am J Med Genet.

[CR25] Chamberlain SJ, Lalande M (2010). Angelman syndrome, a genomic imprinting disorder of the brain. J Neurosci.

[CR26] Charalambous M, da Rocha ST, Ferguson-Smith AC (2007). Genomic imprinting, growth control and the allocation of nutritional resources: consequences for postnatal life. Curr Opin Endocrinol Diabetes Obes.

[CR27] Cheng Y, An Q, Wang J, Wang Y, Dong J, Yin J (2020). RasGRF1 participates in the protective effect of tanshinone IIA on depressive like behaviors of a chronic unpredictable mild stress induced mouse model. Gene.

[CR200] Clinton SM, Vázquez DM, Kabbaj M, Kabbaj MH, Watson SJ, Akil H (2007). Individual differences in novelty-seeking and emotional reactivity correlate with variation in maternal behavior. Horm Behav.

[CR201] Clinton SM, Bedrosian TA, Abraham AD, Watson SJ, Akil H (2010). Neural and environmental factors impacting maternal behavior differences in high- versus low-novelty-seeking rats. Horm Behav.

[CR28] Cohen JL, Glover ME, Pugh PC, Fant AD, Simmons RK, Akil H, Kerman IA, Clinton SM (2015). Maternal style selectively shapes amygdalar development and social behavior in rats genetically prone to high anxiety. Dev Neurosci.

[CR29] Croteau S, Roquis D, Charron MC, Frappier D, Yavin D, Loredo-Osti JC, Hudson TJ, Naumova AK (2005). Increased plasticity of genomic imprinting of Dlk1 in brain is due to genetic and epigenetic factors. Mamm Genome.

[CR30] Curley JP, Barton S, Surani A, Keverne EB (2004). Coadaptation in mother and infant regulated by a paternally expressed imprinted gene. Proc Biol Sci.

[CR31] Davies W, Isles AR, Wilkinson LS (2005). Imprinted gene expression in the brain. Neurosci Biobehav Rev.

[CR32] DeChiara TM, Efstratiadis A, Robertson EJ (1990). A growth-deficiency phenotype in heterozygous mice carrying an insulin-like growth factor II gene disrupted by targeting. Nature.

[CR33] Desai M, Jellyman JK, Han G, Beall M, Lane RH, Ross MG (2014). Maternal obesity and high-fat diet program offspring metabolic syndrome. Am J Obstet Gynecol.

[CR34] Desai M, Li T, Han G, Ross MG (2014). Programmed hyperphagia secondary to increased hypothalamic SIRT1. Brain Res.

[CR35] DeVeale B, van der Kooy D, Babak T (2012). Critical evaluation of imprinted gene expression by RNA-Seq: a new perspective. PLoS Genet.

[CR36] Dockery L, Gerfen J, Harview C, Rahn-Lee C, Horton R, Park Y, Davis TL (2009). Differential methylation persists at the mouse Rasgrf1 DMR in tissues displaying monoallelic and biallelic expression. Epigenetics.

[CR37] Drake NM, Park YJ, Shirali AS, Cleland TA, Soloway PD (2009). Imprint switch mutations at Rasgrf1 support conflict hypothesis of imprinting and define a growth control mechanism upstream of IGF1. Mamm Genome.

[CR38] Duffie R, Ajjan S, Greenberg MV, Zamudio N, Escamilla del Arenal M, Iranzo J, Okamoto I, Barbaux S, Fauque P, Bourc'his D (2014). The Gpr1/Zdbf2 locus provides new paradigms for transient and dynamic genomic imprinting in mammals. Genes Dev.

[CR39] Eclarinal JD, Zhu S, Baker MS, Piyarathna DB, Coarfa C, Fiorotto ML, Waterland RA (2016). Maternal exercise during pregnancy promotes physical activity in adult offspring. FASEB J.

[CR40] Eichenlaub-Ritter U, Adler ID, Carere A, Pacchierotti F (2007). Gender differences in germ-cell mutagenesis and genetic risk. Environ Res.

[CR41] Fernandez-Medarde A (1815). Santos E (2011) the RasGrf family of mammalian guanine nucleotide exchange factors. Biochim Biophys Acta.

[CR42] Fish EW, Shahrokh D, Bagot R, Caldji C, Bredy T, Szyf M, Meaney MJ (2004). Epigenetic programming of stress responses through variations in maternal care. Ann N Y Acad Sci.

[CR43] Fitzpatrick GV, Pugacheva EM, Shin JY, Abdullaev Z, Yang Y, Khatod K, Lobanenkov VV, Higgins MJ (2007). Allele-specific binding of CTCF to the multipartite imprinting control region KvDMR1. Mol Cell Biol.

[CR44] Franklin TB, Mansuy IM (2010). The prevalence of epigenetic mechanisms in the regulation of cognitive functions and behaviour. Curr Opin Neurobiol.

[CR45] Garfield AS, Cowley M, Smith FM, Moorwood K, Stewart-Cox JE, Gilroy K, Baker S, Xia J, Dalley JW, Hurst LD, Wilkinson LS, Isles AR, Ward A (2011). Distinct physiological and behavioural functions for parental alleles of imprinted Grb10. Nature.

[CR46] Garland T, Cadney MD, Waterland RA (2017). Early-life effects on adult physical activity: concepts, relevance, and experimental approaches. Physiol Biochem Zool.

[CR47] Gregg C, Zhang J, Weissbourd B, Luo S, Schroth GP, Haig D, Dulac C (2010). High-resolution analysis of parent-of-origin allelic expression in the mouse brain. Science.

[CR48] Hark AT, Schoenherr CJ, Katz DJ, Ingram RS, Levorse JM, Tilghman SM (2000). CTCF mediates methylation-sensitive enhancer-blocking activity at the H19/Igf2 locus. Nature.

[CR49] Harper KM, Tunc-Ozcan E, Graf EN, Herzing LB, Redei EE (2014). Intergenerational and parent of origin effects of maternal calorie restriction on Igf2 expression in the adult rat hippocampus. Psychoneuroendocrinology.

[CR50] Heijmans BT, Tobi EW, Stein AD, Putter H, Blauw GJ, Susser ES, Slagboom PE, Lumey LH (2008). Persistent epigenetic differences associated with prenatal exposure to famine in humans. Proc Natl Acad Sci U S A.

[CR51] Heim C, Newport DJ, Wagner D, Wilcox MM, Miller AH, Nemeroff CB (2002). The role of early adverse experience and adulthood stress in the prediction of neuroendocrine stress reactivity in women: a multiple regression analysis. Depress Anxiety.

[CR52] Hillis DA, Yadgary L, Weinstock GM, Pardo-Manuel de Villena F, Pomp D, Fowler AS, Xu S, Chan F, Garland T (2020). Genetic basis of aerobically supported voluntary exercise: results from a selection experiment with house mice. Genetics.

[CR53] Hiramatsu L, Kay JC, Thompson Z, Singleton JM, Claghorn GC, Albuquerque RL, Ho B, Ho B, Sanchez G, Garland T (2017). Maternal exposure to Western diet affects adult body composition and voluntary wheel running in a genotype-specific manner in mice. Physiol Behav.

[CR54] Holmes R, Chang Y, Soloway PD (2006). Timing and sequence requirements defined for embryonic maintenance of imprinted DNA methylation at Rasgrf1. Mol Cell Biol.

[CR55] Hoyo C, Murtha AP, Schildkraut JM, Jirtle RL, Demark-Wahnefried W, Forman MR, Iversen ES, Kurtzberg J, Overcash F, Huang Z, Murphy SK (2011). Methylation variation at IGF2 differentially methylated regions and maternal folic acid use before and during pregnancy. Epigenetics.

[CR56] Huang WC, Ferris E, Cheng T, Horndli CS, Gleason K, Tamminga C, Wagner JD, Boucher KM, Christian JL, Gregg C (2017). Diverse non-genetic, allele-specific expression effects shape genetic architecture at the cellular level in the mammalian brain. Neuron.

[CR57] Huang WC, Bennett K, Gregg C (2018). Epigenetic and cellular diversity in the brain through allele-specific effects. Trends Neurosci.

[CR58] Imaizumi Y, Furutachi S, Watanabe T, Miya H, Kawaguchi D, Gotoh Y (2020). Role of the imprinted allele of the Cdkn1c gene in mouse neocortical development. Sci Rep.

[CR59] Isles AR, Wilkinson LS (2000). Imprinted genes, cognition and behaviour. Trends Cogn Sci.

[CR60] Itier JM, Tremp GL, Leonard JF, Multon MC, Ret G, Schweighoffer F, Tocque B, Bluet-Pajot MT, Cormier V, Dautry F (1998). Imprinted gene in postnatal growth role. Nature.

[CR61] Jouvenot Y, Poirier F, Jami J, Paldi A (1999). Biallelic transcription of Igf2 and H19 in individual cells suggests a post-transcriptional contribution to genomic imprinting. Curr Biol.

[CR62] Kalish JM, Jiang C, Bartolomei MS (2014). Epigenetics and imprinting in human disease. Int J Dev Biol.

[CR63] Kelly SA, Nehrenberg DL, Hua K, Gordon RR, Garland T, Pomp D (2010). Parent-of-origin effects on voluntary exercise levels and body composition in mice. Physiol Genomics.

[CR64] Kelly SA, Nehrenberg DL, Hua K, Garland T, Pomp D (2012). Functional genomic architecture of predisposition to voluntary exercise in mice: expression QTL in the brain. Genetics.

[CR65] Kessler MS, Bosch OJ, Bunck M, Landgraf R, Neumann ID (2011). Maternal care differs in mice bred for high vs low trait anxiety: impact of brain vasopressin and cross-fostering. Soc Neurosci.

[CR66] Keverne EB (1996). Genomic imprinting and the differential roles of parental genomes in brain development. Brain Res Dev Brain Res.

[CR67] Kile ML, Fang S, Baccarelli AA, Tarantini L, Cavallari J, Christiani DC (2013). A panel study of occupational exposure to fine particulate matter and changes in DNA methylation over a single workday and years worked in boilermaker welders. Environ Health.

[CR68] Kim J, Perera BPU, Ghimire S (2018). Enhancer-driven alternative promoters of imprinted genes. PLoS ONE.

[CR69] Kinney SM, Chin HG, Vaisvila R, Bitinaite J, Zheng Y, Esteve PO, Feng S, Stroud H, Jacobsen SE, Pradhan S (2011). Tissue-specific distribution and dynamic changes of 5-hydroxymethylcytosine in mammalian genomes. J Biol Chem.

[CR70] Kobayashi H, Yamada K, Morita S, Hiura H, Fukuda A, Kagami M, Ogata T, Hata K, Sotomaru Y, Kono T (2009). Identification of the mouse paternally expressed imprinted gene Zdbf2 on chromosome 1 and its imprinted human homolog ZDBF2 on chromosome 2. Genomics.

[CR71] Kobayashi H, Sakurai T, Sato S, Nakabayashi K, Hata K, Kono T (2012). Imprinted DNA methylation reprogramming during early mouse embryogenesis at the Gpr1-Zdbf2 locus is linked to long cis-intergenic transcription. FEBS Lett.

[CR72] Kochmanski J, Marchlewicz EH, Savidge M, Montrose L, Faulk C, Dolinoy DC (2017). Longitudinal effects of developmental bisphenol a and variable diet exposures on epigenetic drift in mice. Reprod Toxicol.

[CR73] Kostrzewa E, Kas MJ (2014). The use of mouse models to unravel genetic architecture of physical activity: a review. Genes Brain Behav.

[CR74] Kovacheva VP, Mellott TJ, Davison JM, Wagner N, Lopez-Coviella I, Schnitzler AC, Blusztajn JK (2007). Gestational choline deficiency causes global and Igf2 gene DNA hypermethylation by up-regulation of Dnmt1 expression. J Biol Chem.

[CR75] Kravitz SN, Gregg C (2019). New subtypes of allele-specific epigenetic effects: implications for brain development, function and disease. Curr Opin Neurobiol.

[CR76] Krueger F, Andrews SR (2011). Bismark: a flexible aligner and methylation caller for bisulfite-seq applications. Bioinformatics.

[CR77] Landers M, Bancescu DL, Le Meur E, Rougeulle C, Glatt-Deeley H, Brannan C, Muscatelli F, Lalande M (2004). Regulation of the large (approximately 1000 kb) imprinted murine Ube3a antisense transcript by alternative exons upstream of Snurf/Snrpn. Nucleic Acids Res.

[CR78] LaSalle JM, Reiter LT, Chamberlain SJ (2015). Epigenetic regulation of UBE3A and roles in human neurodevelopmental disorders. Epigenomics.

[CR79] Laukoter S, Beattie R, Pauler FM, Amberg N, Nakayama KI, Hippenmeyer S (2020). Imprinted Cdkn1c genomic locus cell-autonomously promotes cell survival in cerebral cortex development. Nat Commun.

[CR80] Lefebvre L, Viville S, Barton SC, Ishino F, Keverne EB, Surani MA (1998). Abnormal maternal behaviour and growth retardation associated with loss of the imprinted gene Mest. Nat Genet.

[CR81] Leighton PA, Saam JR, Ingram RS, Stewart CL, Tilghman SM (1995). An enhancer deletion affects both H19 and Igf2 expression. Genes Dev.

[CR82] Li L, Keverne EB, Aparicio SA, Ishino F, Barton SC, Surani MA (1999). Regulation of maternal behavior and offspring growth by paternally expressed Peg3. Science.

[CR83] Li G, Kohorst JJ, Zhang W, Laritsky E, Kunde-Ramamoorthy G, Baker MS, Fiorotto ML, Waterland RA (2013). Early postnatal nutrition determines adult physical activity and energy expenditure in female mice. Diabetes.

[CR84] Maccani JZ, Koestler DC, Houseman EA, Armstrong DA, Marsit CJ, Kelsey KT (2015). DNA methylation changes in the placenta are associated with fetal manganese exposure. Reprod Toxicol.

[CR85] Maeda T, Higashimoto K, Jozaki K, Yatsuki H, Nakabayashi K, Makita Y, Tonoki H, Okamoto N, Takada F, Ohashi H, Migita M, Kosaki R, Matsubara K, Ogata T, Matsuo M, Hamasaki Y, Ohtsuka Y, Nishioka K, Joh K, Mukai T, Hata K, Soejima H (2014). Comprehensive and quantitative multilocus methylation analysis reveals the susceptibility of specific imprinted differentially methylated regions to aberrant methylation in beckwith-wiedemann syndrome with epimutations. Genet Med.

[CR86] Malisch JL, Saltzman W, Gomes FR, Rezende EL, Jeske DR, Garland T (2007). Baseline and stress-induced plasma corticosterone concentrations of mice selectively bred for high voluntary wheel running. Physiol Biochem Zool.

[CR87] McCarty R (2017). Cross-fostering: elucidating the effects of genexenvironment interactions on phenotypic development. Neurosci Biobehav Rev.

[CR88] McGrath J, Solter D (1984). Completion of mouse embryogenesis requires both the maternal and paternal genomes. Cell.

[CR89] McGreevy KR, Tezanos P, Ferreiro-Villar I, Palle A, Moreno-Serrano M, Esteve-Codina A, Lamas-Toranzo I, Bermejo-Alvarez P, Fernandez-Punzano J, Martin-Montalvo A, Montalban R, Ferron SR, Radford EJ, Fontan-Lozano A, Trejo JL (2019). Intergenerational transmission of the positive effects of physical exercise on brain and cognition. Proc Natl Acad Sci U S A.

[CR90] McNamara MP, Singleton JM, Cadney MD, Ruegger PM, Borneman J, Garland T (2021). Early-life effects of juvenile Western diet and exercise on adult gut microbiome composition in mice. J Exp Biol.

[CR91] Meaney MJ (2001). Maternal care, gene expression, and the transmission of individual differences in stress reactivity across generations. Annu Rev Neurosci.

[CR92] Meek TH, Eisenmann JC, Keeney BK, Hannon RM, Dlugosz EM, Garland T (2014). Effects of early-life exposure to Western diet and wheel access on metabolic syndrome profiles in mice bred for high voluntary exercise. Genes Brain Behav.

[CR93] Millership SJ, Van de Pette M, Withers DJ (2019). Genomic imprinting and its effects on postnatal growth and adult metabolism. Cell Mol Life Sci.

[CR94] Mochida GH, Mahajnah M, Hill AD, Basel-Vanagaite L, Gleason D, Hill RS, Bodell A, Crosier M, Straussberg R, Walsh CA (2009). A truncating mutation of TRAPPC9 is associated with autosomal-recessive intellectual disability and postnatal microcephaly. Am J Hum Genet.

[CR95] Monk D, Arnaud P, Frost J, Hills FA, Stanier P, Feil R, Moore GE (2009). Reciprocal imprinting of human GRB10 in placental trophoblast and brain: evolutionary conservation of reversed allelic expression. Hum Mol Genet.

[CR96] Monk D, Mackay DJG, Eggermann T, Maher ER, Riccio A (2019). Genomic imprinting disorders: lessons on how genome, epigenome and environment interact. Nat Rev Genet.

[CR97] Montrose L, Faulk C, Francis J, Dolinoy DC (2017). Perinatal lead (Pb) exposure results in sex and tissue-dependent adult DNA methylation alterations in murine IAP transposons. Environ Mol Mutagen.

[CR98] Moreland E, Borisov OV, Semenova EA, Larin AK, Andryushchenko ON, Andryushchenko LB, Generozov EV, Williams AG, Ahmetov II (2020). Polygenic profile of elite strength athletes. J Strength Cond Res.

[CR99] Mouallem M, Shaharabany M, Weintrob N, Shalitin S, Nagelberg N, Shapira H, Zadik Z, Farfel Z (2008). Cognitive impairment is prevalent in pseudohypoparathyroidism type Ia, but not in pseudopseudohypoparathyroidism: possible cerebral imprinting of Gsalpha. Clin Endocrinol (oxf).

[CR100] Murphy SK, Adigun A, Huang Z, Overcash F, Wang F, Jirtle RL, Schildkraut JM, Murtha AP, Iversen ES, Hoyo C (2012). Gender-specific methylation differences in relation to prenatal exposure to cigarette smoke. Gene.

[CR101] Murphy SK, Huang Z, Hoyo C (2012). Differentially methylated regions of imprinted genes in prenatal, perinatal and postnatal human tissues. PLoS ONE.

[CR102] Peall KJ, Smith DJ, Kurian MA, Wardle M, Waite AJ, Hedderly T, Lin JP, Smith M, Whone A, Pall H, White C, Lux A, Jardine P, Bajaj N, Lynch B, Kirov G, O'Riordan S, Samuel M, Lynch T, King MD, Chinnery PF, Warner TT, Blake DJ, Owen MJ, Morris HR (2013). SGCE mutations cause psychiatric disorders: clinical and genetic characterization. Brain.

[CR103] Perez JD, Rubinstein ND, Dulac C (2016). New perspectives on genomic imprinting, an essential and multifaceted mode of epigenetic control in the developing and adult brain. Annu Rev Neurosci.

[CR104] Plass C, Shibata H, Kalcheva I, Mullins L, Kotelevtseva N, Mullins J, Kato R, Sasaki H, Hirotsune S, Okazaki Y, Held WA, Hayashizaki Y, Chapman VM (1996). Identification of Grf1 on mouse chromosome 9 as an imprinted gene by RLGS-M. Nat Genet.

[CR105] Proudhon C, Duffie R, Ajjan S, Cowley M, Iranzo J, Carbajosa G, Saadeh H, Holland ML, Oakey RJ, Rakyan VK, Schulz R, Bourc'his D (2012). Protection against de novo methylation is instrumental in maintaining parent-of-origin methylation inherited from the gametes. Mol Cell.

[CR106] Radford EJ, Ferron SR, Ferguson-Smith AC (2011). Genomic imprinting as an adaptative model of developmental plasticity. FEBS Lett.

[CR107] Reik W, Walter J (2001). Evolution of imprinting mechanisms: the battle of the sexes begins in the zygote. Nat Genet.

[CR108] Rhodes JS, Gammie SC, Garland T (2005). Neurobiology of mice selected for high voluntary wheel-running activity. Integr Comp Biol.

[CR109] Robles-Matos N, Artis T, Simmons RA, Bartolomei MS (2021). Environmental exposure to endocrine disrupting chemicals influences genomic imprinting, growth, and metabolism. Genes (basel).

[CR110] Sandovici I, Leppert M, Hawk PR, Suarez A, Linares Y, Sapienza C (2003). Familial aggregation of abnormal methylation of parental alleles at the IGF2/H19 and IGF2R differentially methylated regions. Hum Mol Genet.

[CR111] Saul MC, Majdak P, Perez S, Reilly M, Garland T, Rhodes JS (2017). High motivation for exercise is associated with altered chromatin regulators of monoamine receptor gene expression in the striatum of selectively bred mice. Genes Brain Behav.

[CR112] Schroeder M, Weller A (2010). Anxiety-like behavior and locomotion in CCK1 knockout rats as a function of strain, sex and early maternal environment. Behav Brain Res.

[CR113] Sherwani SI, Khan HA (2015). Role of 5-hydroxymethylcytosine in neurodegeneration. Gene.

[CR114] Shi W, Lefebvre L (2004). Loss-of-imprinting of Peg in mouse interspecies hybrids is correlated with altered growth. Genesis.

[CR115] Shibata H, Yoda Y, Kato R, Ueda T, Kamiya M, Hiraiwa N, Yoshiki A, Plass C, Pearsall RS, Held WA, Muramatsu M, Sasaki H, Kusakabe M, Hayashizaki Y (1998). A methylation imprint mark in the mouse imprinted gene Grf1/Cdc25Mm locus shares a common feature with the U2afbp-rs gene: an association with a short tandem repeat and a hypermethylated region. Genomics.

[CR116] Short AK, Yeshurun S, Powell R, Perreau VM, Fox A, Kim JH, Pang TY, Hannan AJ (2017). Exercise alters mouse sperm small noncoding RNAs and induces a transgenerational modification of male offspring conditioned fear and anxiety. Transl Psychiatry.

[CR117] Soubry A, Murphy S, Huang Z, Murtha A, Schildkraut J, Jirtle R, Wang F, Kurtzberg J, Demark-Wahnefried W, Forman M, Hoyo C (2011). The effects of depression and use of antidepressive medicines during pregnancy on the methylation status of the IGF2 imprinted control regions in the offspring. Clin Epigenetics.

[CR118] Swallow JG, Carter PA, Garland T (1998). Artificial selection for increased wheel-running behavior in house mice. Behav Genet.

[CR119] Swallow JG, Koteja P, Carter PA, Garland T (1999). Artificial selection for increased wheel-running activity in house mice results in decreased body mass at maturity. J Exp Biol.

[CR120] Thorvaldsen JL, Duran KL, Bartolomei MS (1998). Deletion of the H19 differentially methylated domain results in loss of imprinted expression of H19 and Igf2. Genes Dev.

[CR121] Tobi EW, Lumey LH, Talens RP, Kremer D, Putter H, Stein AD, Slagboom PE, Heijmans BT (2009). DNA methylation differences after exposure to prenatal famine are common and timing- and sex-specific. Hum Mol Genet.

[CR122] Tunster SJ, Jensen AB, John RM (2013). Imprinted genes in mouse placental development and the regulation of fetal energy stores. Reproduction.

[CR123] Turan S, Bastepe M (2013). The GNAS complex locus and human diseases associated with loss-of-function mutations or epimutations within this imprinted gene. Horm Res Paediatr.

[CR124] Umeda K, Negishi M, Katoh H (2019). RasGRF1 mediates brain-derived neurotrophic factor-induced axonal growth in primary cultured cortical neurons. Biochem Biophys Rep.

[CR125] Vangeel EB, Izzi B, Hompes T, Vansteelandt K, Lambrechts D, Freson K, Claes S (2015). DNA methylation in imprinted genes IGF2 and GNASXL is associated with prenatal maternal stress. Genes Brain Behav.

[CR126] Vellers HL, Kleeberger SR, Lightfoot JT (2018). Inter-individual variation in adaptations to endurance and resistance exercise training: genetic approaches towards understanding a complex phenotype. Mamm Genome.

[CR127] Wang X, Sun Q, McGrath SD, Mardis ER, Soloway PD, Clark AG (2008). Transcriptome-wide identification of novel imprinted genes in neonatal mouse brain. PLoS ONE.

[CR128] Waterland RA (2014). Epigenetic mechanisms affecting regulation of energy balance: many questions, few answers. Annu Rev Nutr.

[CR129] Waterland RA, Lin JR, Smith CA, Jirtle RL (2006). Post-weaning diet affects genomic imprinting at the insulin-like growth factor 2 (Igf2) locus. Hum Mol Genet.

[CR130] Weaver IC, Cervoni N, Champagne FA, D'Alessio AC, Sharma S, Seckl JR, Dymov S, Szyf M, Meaney MJ (2004). Epigenetic programming by maternal behavior. Nat Neurosci.

[CR131] Xu S, Garland T (2017). A mixed model approach to genome-wide association studies for selection signatures, with application to mice bred for voluntary exercise behavior. Genetics.

[CR132] Xu YQ, Grundy P, Polychronakos C (1997). Aberrant imprinting of the insulin-like growth factor II receptor gene in Wilms’ tumor. Oncogene.

[CR133] Yamasaki K, Joh K, Ohta T, Masuzaki H, Ishimaru T, Mukai T, Niikawa N, Ogawa M, Wagstaff J, Kishino T (2003). Neurons but not glial cells show reciprocal imprinting of sense and antisense transcripts of Ube3a. Hum Mol Genet.

[CR134] Ye X, Kohtz A, Pollonini G, Riccio A, Alberini CM (2015). Insulin like growth factor 2 expression in the rat brain both in basal condition and following learning predominantly derives from the maternal allele. PLoS ONE.

[CR135] Yeshurun S, Short AK, Bredy TW, Pang TY, Hannan AJ (2017). Paternal environmental enrichment transgenerationally alters affective behavioral and neuroendocrine phenotypes. Psychoneuroendocrinology.

[CR136] Yin MM, Wang W, Sun J, Liu S, Liu XL, Niu YM, Yuan HR, Yang FY, Fu L (2013). Paternal treadmill exercise enhances spatial learning and memory related to hippocampus among male offspring. Behav Brain Res.

[CR137] Yoon BJ, Herman H, Sikora A, Smith LT, Plass C, Soloway PD (2002). Regulation of DNA methylation of Rasgrf1. Nat Genet.

[CR138] Yoon B, Herman H, Hu B, Park YJ, Lindroth A, Bell A, West AG, Chang Y, Stablewski A, Piel JC, Loukinov DI, Lobanenkov VV, Soloway PD (2005). Rasgrf1 imprinting is regulated by a CTCF-dependent methylation-sensitive enhancer blocker. Mol Cell Biol.

[CR139] Zamarbide M, Gil-Bea FJ, Bannenberg P, Martinez-Pinilla E, Sandoval J, Franco R, Perez-Mediavilla A (2018). Maternal imprinting on cognition markers of wild type and transgenic Alzheimer’s disease model mice. Sci Rep.

[CR140] Zhang P, Liegeois NJ, Wong C, Finegold M, Hou H, Thompson JC, Silverman A, Harper JW, DePinho RA, Elledge SJ (1997). Altered cell differentiation and proliferation in mice lacking p57KIP2 indicates a role in Beckwith-Wiedemann syndrome. Nature.

[CR141] Zhang P, Rhodes JS, Garland T, Perez SD, Southey BR, Rodriguez-Zas SL (2018). Brain region-dependent gene networks associated with selective breeding for increased voluntary wheel-running behavior. PLoS ONE.

[CR142] Zimprich A, Grabowski M, Asmus F, Naumann M, Berg D, Bertram M, Scheidtmann K, Kern P, Winkelmann J, Muller-Myhsok B, Riedel L, Bauer M, Muller T, Castro M, Meitinger T, Strom TM, Gasser T (2001). Mutations in the gene encoding epsilon-sarcoglycan cause myoclonus-dystonia syndrome. Nat Genet.

