# Supplementary material for: DNA Methylation Analysis of Imprinted Genes in the Cortex and Hippocampus of Cross-Fostered Mice Selectively Bred for Increased Voluntary Wheel-Running
Source: Behav Genet. 2022 Aug 21;52(4-5):281–97. doi: 10.1007/s10519-022-10112-z (PMC9463359; doi:10.1007/s10519-022-10112-z)
Supplement: Supplementary file 3 — Supplementary file3 (DOCX 27 KB) [file 10519_2022_10112_MOESM3_ESM.docx]

## **Supplemental Table 3.** **Type 3 tests of fixed effects by genomic region in the cortex with at least one significant main effect and/or interaction.** Linetype, foster-line, sex, line × fline, line × sex, fline × sex, line × fline × sex were included as terms in all models. Separate models were run for each gene. F-statistic and associated p-values for each gene are reported. Hedges’ g value from select comparisons is also reported (CC vs. HRHR; CC vs. CHR; HRHR vs. HRC). Values ± 0.8 or greater (indicated in red) were deemed large effect sizes. Values between ± 0.5 and ± 0.8 (indicated in blue) were considered as a medium effect size.

| **CORTEX** | | | | | | | | |
| --- | --- | --- | --- | --- | --- | --- | --- | --- |
| **Gene** | **Line** | **Fline** | **Line x Fline** | **Sex** | | **Line x Sex** | **Fline x Sex** | **Line x Fline x Sex** |
| *Mest*  (Exon) | F(1,36)=0.81  p=0.3749 | F(1,36)=0.08  p=0.7801 | F(1,36)=8.43  ***p=0.0063*** | F(1,36)=0.08  p=0.7764 | | F(1,36)=0.02  p=0.8995 | F(1,36)=1.65  p=0.2074 | F(1,36)=0.3  p=0.5890 |
|  | | **L.S. Mean (S.E.)** | **Effect Size** |  | | | | |
| ♀ | CC | 60.95 (1.67) |  | |  | | | |
|  | CHR | 65.14 (1.67) | ***1.12*** (vs. CC) | |  |  |  |  |
|  | HRHR | 63.48 (1.67) | ***0.68*** (vs. CC) | |  |  |  |  |
|  | HRC | 65.16 (1.67) | 0.45 (vs. HRHR) | |  |  |  |  |
| ♂ | CC | 62.38 (1.83) |  | |  |  |  |  |
|  | CHR | 64.73 (1.83) | ***0.57*** (vs. CC) | |  |  |  |  |
|  | HRHR | 61.40 (1.83) | -0.24 (vs. CC) | |  |  |  |  |
|  | HRC | 67.63 (1.83) | ***1.52*** (vs. HRHR) | |  |  |  |  |
|  | | | | | | | | |
| **Gene** | **Line** | **Fline** | **Line x Fline** | **Sex** | | **Line x Sex** | **Fline x Sex** | **Line x Fline x Sex** |
| *Mest*  (Promoter) | F(1,38)=0.08  p=0.7846 | F(1,38)=0.40  p=0.5330 | F(1,38)=3.81  ***p=0.0483*** | F(1,38)=2.39  p=0.1307 | | F(1,38)=0.09  p=0.7675 | F(1,38)=0.5  p=0.4850 | F(1,38)=1.71  p=0.1993 |
|  | | **L.S. Mean (S.E.)** | **Effect Size** |  | | | | |
| ♀ | CC | 54.90 (1.30) |  | |  | | | |
|  | CHR | 54.24 (1.30) | -0.28 (vs. CC) | |  |  |  |  |
|  | HRHR | 53.65 (1.30) | -0.43 (vs. CC) | |  |  |  |  |
|  | HRC | 55.53 (1.30) | ***0.65*** (vs. HRHR) | |  |  |  |  |
| ♂ | CC | 51.81 (1.43) |  | |  | | | |
|  | CHR | 54.97 (1.30) | ***1.03*** (vs. CC) | |  |  |  |  |
|  | HRHR | 51.34 (1.43) | -0.15 (vs. CC) | |  |  |  |  |
|  | HRC | 54.35 (1.30) | ***0.99*** (vs. HRHR) | |  |  |  |  |
|  | | | | | | | | |
| **Gene** | **Line** | **Fline** | **Line x Fline** | **Sex** | | **Line x Sex** | **Fline x Sex** | **Line x Fline x Sex** |
| *Sgce*  (Intron) | F(1,38)=0.09  p=0.7604 | F(1,38)=0.27  p=0.6065 | F(1,38)=0.11  p=0.7410 | F(1,38)=0.46  p=0.5032 | | F(1,38)=0.92  p=0.3446 | F(1,38)=7.66  ***p=0.0087*** | F(1,38)=0.27  p=0.6076 |
|  | | **L.S. Mean (S.E.)** | **Effect Size** |  | | | | |
| ♀ | CC | 55.74 (1.16) |  | |  | | | |
|  | CHR | 57.48 (1.16) | ***0.67*** (vs. CC) | |  |  |  |  |
|  | HRHR | 57.09 (1.16) | ***0.52*** (vs. CC) | |  |  |  |  |
|  | HRC | 55.04 (1.16) | ***-0.79*** (vs. HRHR) | |  |  |  |  |
| ♂ | CC | 57.40 (1.27) |  | |  | | | |
|  | CHR | 55.35 (1.16) | ***-0.75*** (vs. CC) | |  |  |  |  |
|  | HRHR | 55.70 (1.27) | ***-0.60*** (vs. CC) | |  |  |  |  |
|  | HRC | 59.18 (1.16) | ***1.28*** (vs. HRHR) | |  |  |  |  |

## **Supplemental Table 4.** **Type 3 tests of fixed effects by genomic region in the hippocampus with at least one significant main effect and/or interaction.** Linetype, foster-line, sex, line × fline, line × sex, fline × sex, line × fline × sex were included as terms in all models. Separate models were run for each gene. F-statistic and associated p-values for each gene are reported. Hedges’ g value from select comparisons is also reported (CC vs. HRHR; CC vs. CHR; HRHR vs. HRC). Values ± 0.8 or greater (indicated in red) were deemed large effect sizes. Values between ± 0.5 and ± 0.8 (indicated in blue) were considered a medium effect size.

| **HIPPOCAMPUS** | | | | | | | | |
| --- | --- | --- | --- | --- | --- | --- | --- | --- |
| **Gene** | **Line** | **Fline** | **Line x Fline** | **Sex** | | **Line x Sex** | **Fline x Sex** | **Line x Fline x Sex** |
| *Peg3*  (Intron) | F(1,38)=0.3  p=0.5848 | F(1,38)=1.66  p=0.2052 | F(1,38)=8.4  ***p=0.0062*** | F(1,38)=1.26  p=0.2687 | | F(1,38)=0.23  p=0.6344 | F(1,38)=0.31  p=0.5782 | F(1,38)=1.00  p=0.3227 |
|  | | **L.S. Mean (S.E.)** | **Effect Size** |  | | | | |
| ♀ | CC | 68.88 (1.46) |  | |  | | | |
|  | CHR | 68.67 (1.46) | -0.06 (vs. CC) | |  |  |  |  |
|  | HRHR | 66.58 (1.46) | ***-0.70*** (vs. CC) | |  |  |  |  |
|  | HRC | 67.82 (1.46) | 0.38 (vs. HRHR) | |  |  |  |  |
| ♂ | CC | 63.53 (1.60) |  | |  |  |  |  |
|  | CHR | 69.65 (1.46) | ***1.79*** (vs. CC) | |  |  |  |  |
|  | HRHR | 64.40 (1.60) | 0.24 (vs. CC) | |  |  |  |  |
|  | HRC | 66.59 (1.46) | ***0.64*** (vs. HRHR) | |  |  |  |  |
|  | | | | | | | | |
| **Gene** | **Line** | **Fline** | **Line x Fline** | **Sex** | | **Line x Sex** | **Fline x Sex** | **Line x Fline x Sex** |
| *Peg3*  (UTR) | F(1,38)=2.48  p=0.1233 | F(1,38)=0.01  p=0.9394 | F(1,38)=1.29  p=0.2626 | F(1,38)=0.86  p=0.3591 | | F(1,38)=5.26  ***p=0.0274*** | F(1,38)=0.11  p=0.7433 | F(1,38)=0.26  p=0.6103 |
|  | | **L.S. Mean (S.E.)** | **Effect Size** |  | | | | |
| ♀ | CC | 62.97 (1.71) |  | |  | | | |
|  | CHR | 64.06 (1.71) | 0.29 (vs. CC) | |  |  |  |  |
|  | HRHR | 64.18 (1.71) | 0.32 (vs. CC) | |  |  |  |  |
|  | HRC | 64.64 (1.71) | 0.12 (vs. HRHR) | |  |  |  |  |
| ♂ | CC | 64.44 (1.87) |  | |  |  |  |  |
|  | CHR | 65.98 (1.71) | 0.38 (vs. CC) | |  |  |  |  |
|  | HRHR | 59.14 (1.87) | ***-1.27*** (vs. CC) | |  |  |  |  |
|  | HRC | 61.69 (1.71) | ***0.64*** (vs. HRHR) | |  |  |  |  |
|  | | | | | | | | |
| **Gene** | **Line** | **Fline** | **Line x Fline** | **Sex** | | **Line x Sex** | **Fline x Sex** | **Line x Fline x Sex** |
| *Sgce*  (Intron) | F(1,38)=0.41  p=0.5248 | F(1,38)=0.52  p=0.4746 | F(1,38)=0.01  p=0.9242 | F(1,38)=0.04  p=0.8390 | | F(1,38)=0.12  p=0.7345 | F(1,38)=2.53  p=0.1197 | F(1,38)=6.26  ***p=0.0168*** |
|  | | **L.S. Mean (S.E.)** | **Effect Size** |  | | | | |
| ♀ | CC | 58.15 (1.87) |  | |  | | | |
|  | CHR | 56.66 (1.87) | -0.36 (vs. CC) | |  |  |  |  |
|  | HRHR | 59.37 (1.87) | 0.29 (vs. CC) | |  |  |  |  |
|  | HRC | 59.26 (1.87) | 0.03 (vs. HRHR) | |  |  |  |  |
| ♂ | CC | 59.56 (2.04) |  | |  |  |  |  |
|  | CHR | 57.24 (1.87) | ***-0.53*** (vs. CC) | |  |  |  |  |
|  | HRHR | 59.08 (2.04) | -0.11 (vs. CC) | |  |  |  |  |
|  | HRC | 57.63 (2.04) | -0.32 (vs. HRHR) | |  |  |  |  |
