# Supplementary material for: DNA Methylation Analysis of Imprinted Genes in the Cortex and Hippocampus of Cross-Fostered Mice Selectively Bred for Increased Voluntary Wheel-Running
Source: Behav Genet. 2022 Aug 21;52(4-5):281–97. doi: 10.1007/s10519-022-10112-z (PMC9463359; doi:10.1007/s10519-022-10112-z)
Supplement: Supplementary file 4 — Supplementary file4 (DOCX 16 KB) [file 10519_2022_10112_MOESM4_ESM.docx]

**Supplemental Table 1. Percent methylation levels for CpG sites across the entire genomic region for each gene.** p-values represent differences in the L.S. means between the in-fostered and cross-fostered groups and include main effects and interactions (line × foster-line, line × sex, foster-line × sex, line × foster-line × sex).

**Supplemental Table 2. Percent methylation levels for CpG sites within distinct genomic regions for each gene.** p-values represent differences in the L.S. means between the in-fostered and cross-fostered groups and include main effects and interactions (line × foster-line, line × sex, foster-line × sex, line × foster-line × sex).

## **Supplemental Table 3.** **Type 3 tests of fixed effects by genomic region in the cortex with at least one significant main effect and/or interaction.** Line, foster-line, sex, line × fline, line × sex, fline × sex, line × fline × sex were included as terms in all models. Separate models were run for each gene. F-statistic and associated p-values for each gene are reported. Hedges’ g value from select comparisons is also reported (CC vs. HRHR; CC vs. CHR; HRHR vs. HRC). Values ± 0.8 or greater (indicated in red) were viewed as large effect sizes. Values between ± 0.5 and ± 0.8 (indicated in blue) were considered as a medium effect size.

## **Supplemental Table 4.** **Type 3 tests of fixed effects by genomic region in the hippocampus with at least one significant main effect and/or interaction.** Line, foster-line, sex, line × fline, line × sex, fline × sex, line × fline × sex were included as terms in all models. Separate models were run for each gene. F-statistic and associated p-values for each gene are reported. Hedges’ g value from select comparisons is also reported (CC vs. HRHR; CC vs. CHR; HRHR vs. HRC). Values ± 0.8 or greater (indicated in red) were viewed as large effect sizes. Values between ± 0.5 and ± 0.8 (indicated in blue) were considered a medium effect size.
